# Multiparametric analysis in the peripheral blood of Giant Cell Arteritis and Polymyalgia Rheumatica patients at the early phases of steroid treatment reveals changes in cell subpopulations and lipid mediators: a preliminary study

**DOI:** 10.3389/fimmu.2025.1594263

**Published:** 2025-06-04

**Authors:** Dimitris Anastasios Palamidas, Maria Papadaki, Nikolaos Paschalidis, Eleftherios Pavlos, Loukas Chatzis, Panagiota Palla, Ourania D. Argyropoulou, Dionysios Prevezanos, Marc Dubourdeau, Konstantinos Kambas, Ioanna Evdokia Galani, Andreas V. Goules, Evangelos Andreakos, Athanasios G. Tzioufas

**Affiliations:** ^1^ Department of Pathophysiology, and Joint Academic Rheumatology Program, School of Medicine, National and Kapodistrian University of Athens, Athens, Greece; ^2^ Laboratory of Immunobiology, Center for Clinical, Experimental Surgery and Translational Research, Biomedical Research Foundation of the Academy of Athens, Athens, Greece; ^3^ Biomedical Research Foundation of the Academy of Athens, Athens, Greece; ^4^ Division of Basic Sciences, University of Crete Medical School, Heraklion, Greece; ^5^ Research Institute for Systemic Autoimmune Diseases, Athens, Greece; ^6^ Second Propedeutic Department of Surgery, National and Kapodistrian University of Athens, Athens, Greece; ^7^ Ambiotis SAS, Toulouse, France; ^8^ Laboratory of Molecular Genetics, Department of Immunology, Hellenic Pasteur Institute, Athens, Greece

**Keywords:** GCA, PMR, steroids, CyTOF, lipidomics

## Abstract

**Introduction:**

Giant Cell Arteritis (GCA) and Polymyalgia Rheumatica (PMR) are autoimmune/autoinflammatory disorders presenting as acute inflammatory responses and are highly responsive to steroids. In this report, we aim to decipher the immune landscape including immune cell subpopulations, plasma cytokines, and small lipid mediators (LMs) at the very early stages of steroid treatment initiation in 4 distinct time points at: 0h (T1), 48h (T2), 96h (T3), and 24 weeks (T4).

**Patients and methods:**

Serum, plasma and peripheral blood mononuclear cells (PBMCs) were collected prospectively from 8 GCA and 6 PMR newly diagnosed patients. Sixteen healthy individuals served as controls (HC). Deep immunophenotyping by CyTOF was performed in PBMCs at T1-T3. A multiplex Luminex assay measured serum levels of 21 cytokines at T1 and T3. Levels of lipid mediators (LMs) were evaluated at T1, T3 and T4 with the LC-MS/MS method.

**Results:**

Total CD8+ T cells and DCs were decreased within 48–96 hours, following steroid treatment, while B-cells were increased at 48 hours. Further analysis of immune subpopulations containing the major cell types revealed different frequencies of distinct CD8+, CD4+, DCs, and B cell subtypes. Out of 21 cytokines/chemokines evaluated, only ITAC levels were decreased at T3. The ratio of pro/anti-inflammatory LMs was high at T1 in patients with either PMR or GCA. However, 6 months after steroid treatment it returned to normal in PMR patients, but remained high in GCA patients, providing the only discriminatory element between the two diseases.

**Conclusion:**

The rapid clinical improvement of GCA and PMR patients, following steroid treatment, is associated with immune cell type alterations, but it is poorly associated with plasma cytokine levels. Small lipid mediators can differentiate GCA and PMR patients. The persistently elevated levels of pro-inflammatory LMs might be related to the underlying residual tissue inflammation described in GCA. These preliminary results suggest that further studies in a larger patient cohort are required to validate these findings.

## 1 Introduction

Giant cell arteritis (GCA) and polymyalgia rheumatica (PMR) are rare autoimmune/autoinflammatory diseases affecting mainly individuals more than 50 years old. These conditions are characterized by robust and acute inflammatory responses, with IL-6 playing a central role (1). Glucocorticoids (GC) remain the cornerstone of treatment with an immediate symptomatic effect, clinically apparent even within 48–72 hours after administration.

Even though GCA and PMR share common inflammatory features seen in the peripheral blood, they are characterized by distinct underlying pathophysiologic mechanisms in the affected tissue. In GCA-positive temporal artery biopsies (TABs), key immune components include Th1 and Th17 lymphocytes which predominate *in situ*, producing IFN-γ and IL-17 respectively, inducing the recruitment of monocytes and neutrophils, amplifying the inflammatory cascade and, eventually, tissue injury. Macrophages contribute via granuloma formation, while dendritic cells reside in the adventitia and activate naïve T cells. B cells are also observed in the context of GCA, but their exact role in the inflamed tissue is not entirely delineated (2). Different studies have explored the frequencies of major immune cell types in the peripheral blood of GCA patients during active disease and in remission after long-term treatment (3–5). However, there are no studies investigating the full immune landscape in the peripheral blood and the inflamed tissue to draw a detailed picture of the underlying pathogenetic mechanism. Little is known about the peripheral blood mononuclear cell (PBMC) populations within the first days of steroid treatment. On the other hand, in PMR, where inflamed tissues such as bursae and tendon sheaths are less accessible for analysis, most pathophysiologic insights come from peripheral blood studies. Systemic inflammation is primarily driven by circulating monocytes/macrophages producing key pro-inflammatory cytokines, with IL-6 being in a leading position (6). CD4+ T cells also contribute, but the exact subsets are less prominent than in GCA, and the role of B cells, remains similarly undefined (7).

A critical yet understudied component of acute inflammation in both diseases is the role of bioactive lipid mediators (LMs). Bioactive lipids constitute a large group of molecules that modulate the initiation and resolution of inflammation. They encompass the pro-inflammatory eicosanoids (e.g., leukotrienes, prostaglandins, and thromboxanes) formed by omega-6 polyunsaturated fatty acids (n-6 PUFA), as well as the omega-3 fatty acid (n-3 PUFA) derivatives mediating the resolution of inflammation (8, 9).

Both PMR and GCA exhibit profound steroid responsiveness, particularly in the early disease phases, with glucocorticoids reducing cytokine production and immune cell activation (10, 11). However, the dynamic changes in circulating immune cells, cytokine levels, and bioactive lipid alterations after the initiation of GC treatment and until the initially observed clinical response, have not been investigated. Understanding these early transitions from active to inactive disease states may provide valuable clinical insights into “sensitive to change” inflammatory biomarkers relevant to the pathogenetic operating mechanisms of the disease. Hereafter, we present the results from our preliminary proof of concept study aiming to a) decipher the immune landscape of GCA and PMR patients during diagnosis, and b) investigate the changes in immune cell subpopulations, using deep peripheral blood cell immunophenotyping, multiplex cytokine analysis, and targeted lipidomics in sequential samples of GCA and PMR patients at the early phases of steroid treatment (0, 48h and 96h post-treatment).

## 2 Patients and methods

### 2.1 Patients

Fourteen newly diagnosed patients, 8 with GCA and 6 with PMR who were diagnosed and followed up at the Rheumatology outpatient clinic of the Department of Pathophysiology in Laiko General Hospital, School of Medicine, University of Athens were prospectively recruited between 2021 and 2023. All patients had to fulfill the 1990 American College of Rheumatology/EULAR classification criteria for GCA and the 2012 American College of Rheumatology (ACR)/European Alliance of Associations for Rheumatology (EULAR) classification criteria for PMR (12, 13). GCA patients with positive vascular findings on 18-FDG-PET/CT were defined as having Large Vessel Vasculitis (LVV)-GCA (n= 6). Two patients had only cranial disease (GCA) with positive temporal artery biopsy and negative 18-FDG-PET/CT vascular findings. For patients with PMR diagnosis (n=6), the exclusion of underlying vasculitis was based on a negative temporal artery biopsy and negative 18-FDG-PET/CT scan findings in the arterial tree. Active disease for GCA and PMR patients was defined as the presence of both clinical symptoms and increased acute phase reactants (ESR > 20 mm/h and CRP > 5 mg/L). The opposite definition was applied to define disease remission. All participants were in complete remission after 6 months of treatment. Patients received no DMARD therapy during the study period.

The Ethics Committee of the School of Medicine, National and Kapodistrian University of Athens, Greece, approved the study (protocol no. 1718016656). All participants gave written informed consent before enrollment.

### 2.2 Study design

**Figure 1:**
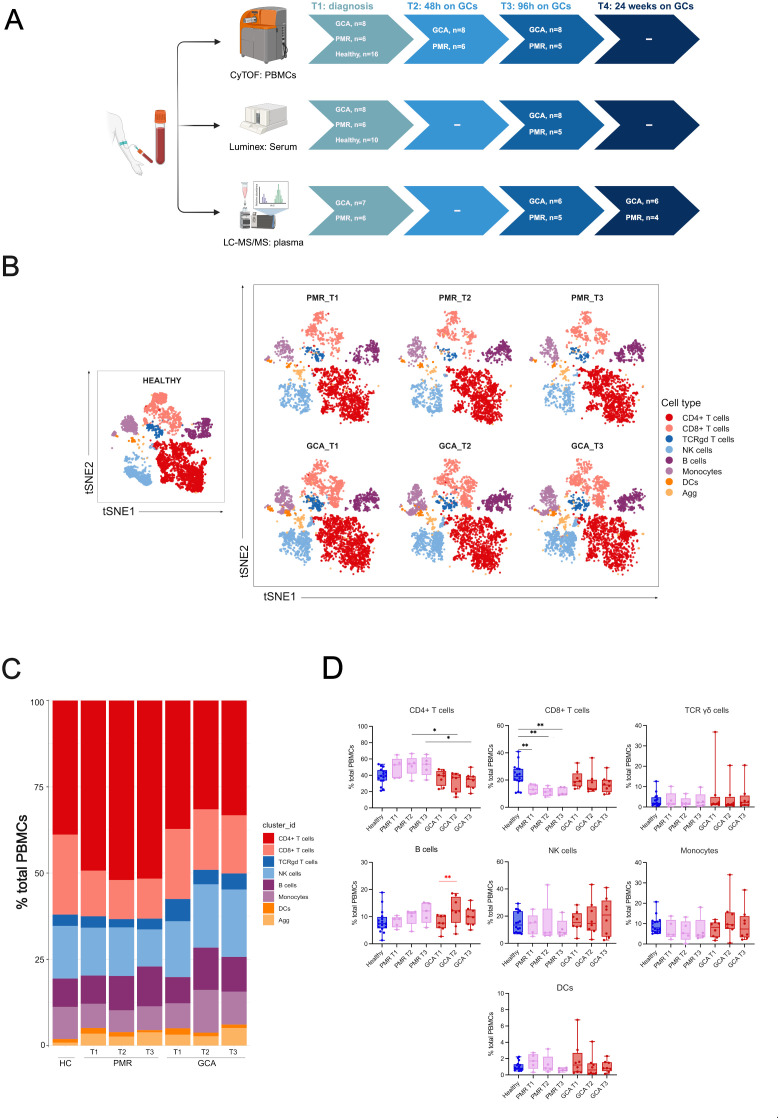
Experimental design and CyTOF clustering of the major immune cell types. **(A)** Schematic representation of the experimental design. Peripheral blood samples were drawn during active disease and before steroid administration (T1), 48 hours after steroid administration (T2), 96 hours after steroid administration (T3), and 6 months after treatment during disease remission (T4). Cytometry by time-of-flight (CyTOF) was performed at T1-T3, Luminex analysis for the quantification of 21 cytokine and chemokine serum levels was performed at T1 and T3 and Lipidomic analysis was performed at T1, T3 and T4 (6 months), **(B)** t-SNE plot visualizing cluster assignments of main cell types in healthy individuals (n=16), PMR (n=6), and GCA patients (n=8) at 3-time points (T1, T2, T3). **(C)** The composition of the major immune populations within total PBMCs (CD45+ immune cells) in healthy individuals, PMR, and GCA patient samples is represented by vertical bars; the length of the colored segment represents the proportion of cells as a percentage of PBMCs. Colors represent different populations within PBMCs, **(D)** The frequencies of major immune cell populations were calculated as the percentage of total PBMCs. Data are presented as box plots with dots showing individual patient measurements. P-values were determined by the Kruskal-Wallis method and adjusted for multiple comparisons by Dunn’s *post hoc* test with Bonferroni correction for between-group comparisons. The Friedman test was followed for one-way repeated analysis between time points in each group. For visualization purposes, the statistical significance is depicted with black asterisks for between-group analysis (Kruskal Wallis test) and with red asterisks for intra-group analysis (Friedman Test). *P<0.05, **P<0.01. The first quartile and the third quartile are shown in the lower and upper horizontal lines, respectively. The horizontal line in the boxes represents the median value. Panel (A) has been created in BioRender. Palamidas, D. (2025) https://BioRender.com/j09s018. PBMCs, peripheral blood mononuclear cells; DCs, dendritic cells; NK, natural killer cells; Agg, aggregates.

A total of 41 PBMC samples from GCA (n=8) and PMR (n=6) patients were selected for Cytometry by Time of Flight (CyTOF) analysis at three time points (T1= diagnosis, T2 = 48h after GC treatment, and T3 = 96h after GC treatment). One PMR patient was unable to participate in the T3 time point. During the same sampling, sera were obtained for Luminex analysis of cytokine/chemokine levels at T1 and T3 (n=41). Lipidomic analysis was performed in T1, T3, and T4 (after 6 months of GC treatment initiation) time points in GCA (n=6) and PMR (n=4) patients’ EDTA plasma samples. Four patients were excluded due to low sample quantity in at least one of the time points tested. A healthy control group (n=16) was also included, matched according to mean age and gender with the patient cohort. CyTOF data from healthy control individuals were selected from previous studies that used the same CyTOF panel and were run on the same CyTOF facility (14, 15). Samples from 10 healthy individuals were included from one study of our laboratory (14), while 6 healthy individual samples were retrieved from the study by Vakrakoy et al. (15) and are available at FlowRepository (http://flowrepository.org/id/FR-FCM-Z5NY) under the following file names AV01.fcs, AV02.fcs, AV03.fcs, AV04.fcs, AV19.fcs, AV20.fcs. Serum and plasma samples were available for Luminex and lipidomics analysis, respectively, for 10 out of 16 healthy individuals. A schematic representation of the study design is presented in **Figure 1A**.

### 2.3 Mass cytometry

In-depth analysis of peripheral blood mononuclear cells was performed by CyTOF with the Maxpar Direct Immunophenotyping Assay (MDIPA) (Standard Biotools, San Francisco, CA) containing a cocktail of 30 pre-conjugated antibodies in a lyophilized form. A complete list of antibodies and conjugated metal isotopes included in the MDIPA kit is presented in **Supplementary Table 1**. PBMCs were isolated from 10 ml whole peripheral blood by density gradient centrifugation using Lymphosep (CatNo: LM-T1702, Biosera, France) according to standard protocols and up to 10^7^ cells were frozen in freezing medium (90% Fetal Bovine Serum, 10% DMSO) at -80°C for 6 months before analysis. For staining with MDIPA kit, PBMCs were thawed in prewarmed RPMI supplemented with 10% FBS, and stained following the manufacturer’s instructions. Cells were stained for live-dead cell discrimination with 1 μM Cisplatin Cell-ID™ (Standard Biotools, San Francisco, CA) and blocked for unspecific antibody binding with Human TruStain FcX (Biolegend). Cells were stained in DNA intercalator solution (1:1000 dilution of 125 μM Cell-ID™ Intercalator-Ir), in Maxpar Fix and Perm buffer (Standard BioTools, San Francisco, CA). Cells were washed with CSB buffer and Cell Acquisition Solution (CAS) the following day. Immediately before the acquisition, cells were resuspended with EQ Passport beads (1:10 dilution). To maximize data quality, the acquisition rate on the Helios™ system (Standard BioTools, South San Francisco, CA, USA) was maintained at a rate of 350 to 400 events/s. Acquired data were normalized using Passport beads (Standard BioTools method) with CyTOF software (version 10.7.1014). Before analysis, we performed data clean-up for Gaussian parameters, and live singlet cell events were used for downstream analyses. Data cleanup, sample quality check, and batch effect control were performed according to previous reports of the CyTOF Laboratory at BRFAA (15).

### 2.4 Luminex assay

Serum samples were collected from patients and healthy individuals and subsequently frozen at −80 °C until further analysis. The MILLIPLEX MAP Human High-Sensitivity T cell Panel (Merck Millipore) was employed for quantifying an analyte panel of 21 cytokines and chemokines by Luminex Assay. IFN-γ, TNF-α, GM-CSF, IL-1β, IL-2, IL-4, IL-5, IL-6, IL-7, IL-8, IL-10, IL-12 (p70), IL-13, IL-17A, IL-21, IL-23, MIP-1α (CCL3), MIP-1β (CCL4), MIP-3α (CCL20), I-TAC (CXCL11) and Fractalkine (CX3CL1). The assay was performed according to the manufacturer’s protocol for serum samples, utilizing recommended sample dilutions and standard curve concentrations (Merck Millipore). Samples were analyzed on a Luminex 200 System using Luminex xPonent v.3.1 software according to the manufacturer’s instructions (Merck Millipore).

### 2.5 Lipidomic analysis

A Liquid Chromatography-tandem Mass Spectrometry method (LC-MS/MS) was used for the quantification of low-level PUFA metabolites. The extraction protocol and LC-MS/MS analysis were performed by AMBIOTIS SAS (Toulouse, France) using a method adapted from Le Faouder et al. (16), by using 100-1000 µL of plasma. Briefly, plasma was mixed with 400 µL of methanol containing BHT (2.6-Di-tert-butyl-4-methylphenol) at 1mM, supernatant collected and spiked with internal standards before loading on HLB oasis cartridges for lipid extraction. After different washings, lipids were eluted with methylformate (MeFor) and methanol (MeOH). Solvent was evaporated under N2 and the residues were recovered in MeOH/H2O (20 µL) to be subjected to LC/MS analysis. Mass spectrometry was conducted using a scheduled Multiple Reaction Monitoring mode on a 6500 + QTRAP (Sciex) apparatus equipped with an electrospray ionization source in negative mode. The sample was injected beforehand into the Exion LCAD U-HPLC system (Sciex) and lipids were separated on a KINETEX C18 column (100 x 2.1 mm; 1.7 µm, Phenomenex) using a binary gradient of H_2_O+0.1% HCOOH/MeOH+0.1% HCOOH. The LC method was optimized to obtain a rapid and accurate separation of 24 molecules, with a pg/mL detection sensitivity in the 20µL of recovery. MRM, retention times and limits of detection (LOQ) are shown in supplemental data. The lipids were identified in the matrix by matching the retention time of the standard injected into the solvent and the blank solvent was injected to determine the baseline of detection. Metabolites were considered as detectable when their peaks were over the baseline. The calibration curve was obtained using the area ratio between the pure standard and the internal standard, injected into the injection solvent. The calibration model was linear with a weighting of 1/X (X=nominal concentrations). Processing was performed with the Sciex OS MQ software. The average of the median of the well-characterized pro-inflammatory LMs: prostaglandins (PGE2, 6k-PGF1a), thromboxane (TXB2), leukotrienes (LTB4) to the average of the median of well-known specialiazed pro-resolving mediators (SPMs): lipoxins (LXA4, LXB4), maresins (Mar2), protectins (PDX, PD1), resolvins D (RvD1-5) and E (RvE1, RvE2, RvE4) series was plotted per patient group.


$$Ratio=\frac{average\left(medianPGE2,\;median6k-PGF1a,\;medianTXB2,\;medianLTB4\right)}{average\begin{array}{c} (medianLXA4,\;medianLXB4,\;medianMar2,\;medianPDX,\;median\\ PD1,\;medianRvD1,\;medianRvD2,\;medianRvD3,\;medianRvD4\\ medianRvD5,\;medianRvE1,\;medianRvE2,\;medianRvE4) \end{array}}$$

### 2.6 Data analysis

Flow cytometry standard (FCS) files were normalized using EQ beads and concatenated. Then files were de-barcoded using the barcode key file (Key_Cell-ID_20-Plex_Pd.csv) in the Fluidigm acquisition software (v. 6.7.1014). Clean-up gates for live single cells and elimination of non-cell signals were manually conducted using the web-plat software, Cytobank (v.9.1). Data were analyzed using a previously described R-based pipeline (17). Briefly, neutrophils were excluded to focus on the remaining cell populations. The data were imported using the flowCore and CATALYST packages, and pre-processing steps included compensation, transformation, and clustering with FlowSOM. Dimensionality reduction was performed using t-distributed stochastic neighbor embedding (t-SNE) to visualize cell populations. Cell type percentages were calculated for each sample, and boxplots were generated to compare proportions across conditions. For t-SNE visualizations, cells were colored by their assigned cluster IDs, and subsets of cells were faceted by condition or timepoint to highlight differences between groups. Differential abundance analysis of clusters and states was performed using the diffcyt package. Statistical significance was set at an FDR threshold of 0.05.

### 2.7 Statistical analysis

Data are presented as medians with the respective interquartile range (IQR) values. The distributions of variables were checked for normality by the Shapiro–Wilk normality test. For non-parametric continuous variables and paired comparisons, the Wilcoxon signed-rank test was used for statistical analysis. A two-tailed p < 0.05 was considered to indicate statistical significance. For non-parametric continuous variables and unpaired comparisons between two groups, the Mann-Whitney test was used. A two-tailed p < 0.05 was considered to indicate statistical significance. For comparisons among multiple groups and for each time point, a non-parametric Kruskal–Wallis test was used followed by Dunn’s *post hoc* test with Bonferroni correction. In cases where intra-group comparisons were made for more than 2 time points, the Friedman test was used for one-way repeated analysis. Analysis was conducted using GraphPad Prism software for Windows (version 10.1.1) (GraphPad Software, San Diego, CA, USA).

## 3 Results

### 3.1 Deep immunophenotyping shows altered immune cell subsets in PMR and GCA patients at the very early phases of steroid treatment

To characterize the immune cell landscape in GCA and PMR patients at the early phases of steroid treatment, we employed CyTOF technology in PBMC samples (n=41) at three time points: 0 (T1), 48h (T2), and 96h (T3) after the initiation of GC treatment. We also included CyTOF data from age and sex-matched healthy individuals (n=16) (**Figure 1A**). A heatmap of the median markers’ expression in all PBMC samples tested and healthy individual data, along with marker annotation of immune cell sub-phenotypes, are presented in **Supplementary Figure 1** and **Supplementary Table 2**, respectively. The demographic, laboratory, and clinical data of patients along with the cumulative steroid dose they received, are described in **Table 1**. t-SNE analysis was performed on all CD45+ cells to determine the major immune cell types within the PBMC fragment in GCA patients, PMR patients, and healthy individuals per time point (T1-T3) (**Figure 1B**). Results indicated a predominance of CD4+ and CD8+ T cells in PBMCs of all samples tested (**Figure 1C**).

**Table 1:** Patients’ characteristics.

Characteristics	LVV/GCA (N=8)^1^	PMR (N=6)
Age (median, years)	72	78
Gender (Female%, N)	75% (6/8)	66,7% (4/6)
ESR (mm/h) in T1 (median)	71	78
ESR (mm/h) in T4 (median)	10,5	12
CRP (mg/l) in T1 (median)	34,5	32
CRP (mg/l) in T4 (median)	2,3	2,35
AH (%, N)	37,5% (3/8)	33,3% (2/6)
DM (%, N)	0% (0/8)	0% (0/6)
Dyslipidemia (%, N)	50% (4/8)	50% (3/6)
PMR symptoms (%, N)	37,5% (3/8)	100% (6/6)
BP lowering drugs (%, N)	37,5% (3/8)	33,3% (2/6)
Glucose lowering drugs (%, N)	0% (0/8)	0% (0/6)
Lipid modifying drugs (%, N)	37,5% (3/8)	33,3% (2/6)
Median starting GC dose T1 (prednisone) (mg), range	40(40-50)	25 (10-40)
Median GC dose at T4 (mg), range	12,5 (7,5-30)	8 (1,3-20)
Cumulative dose of prednisone (mg)	2852	1647
Antiplatelet Tx (%, N)	25% (2/8)	16,6% (1/6)

LVV, Large Vessel Vasculitis; GCA, Giant Cell Arteritis; PMR, Polymyalgia Rheumatica; BP, Blood Pressure; AH, Arterial Hypertension; DB, Diabetes Mellitus; CRP, C-reactive protein; ESR, erythrocyte sedimentation rate; GC, glucocorticoids; Tx, treatment. ^1^All patients in this group had a positive temporal artery biopsy; 6 patients had LVV with positive 18-FDG-PET/CT.

After between-group comparisons, GCA patients exhibited a statistically significant decrease in CD4+ T cell frequencies compared to PMR patients at T2 and T3 (**Figure 1D**). Moreover, PMR patients exhibited a statistically significant decrease in CD8+ T cell frequencies compared to healthy individuals in all time points tested (**Figure 1D**). No further differences were observed in the other major immune cell types after between-group comparisons.

Within-group comparisons were made to depict the changes in major immune cell types over time. Differences in the kinetics of only B cells in the GCA group were observed. B cells were increased from T1 to T2 (**Figure 1D**). No further kinetic changes were observed in the other major immune cell types for each group examined.

We also sought to identify differences in immune cell subsets/subphenotypes, between healthy individuals with GCA or PMR patients. Several differences were observed between healthy individuals and the two disease groups, as presented in **Supplementary Figure 2**. Firstly, CXCR5+ B active cell, mDC, and CD56+ NK cell frequencies were increased in GCA and PMR patients at T1 (**Supplementary Figure 2A**), while pDC and NKT cell frequencies were decreased in both groups at T1 (**Supplementary Figure 2B**). Secondly, B active cell frequencies were increased in the PMR group at T1 (**Supplementary Figure 2C**). Lastly, non-classical monocyte frequencies were decreased in GCA patients at T1 (**Supplementary Figure 2D**), while classical monocyte, HLDR+ CD4+ T cell, CD4+ terminal effector cell, and CD8+ TEMRA cell frequencies were increased in GCA patients at T1 (**Supplementary Figure 2E**).

**Figure 2:**
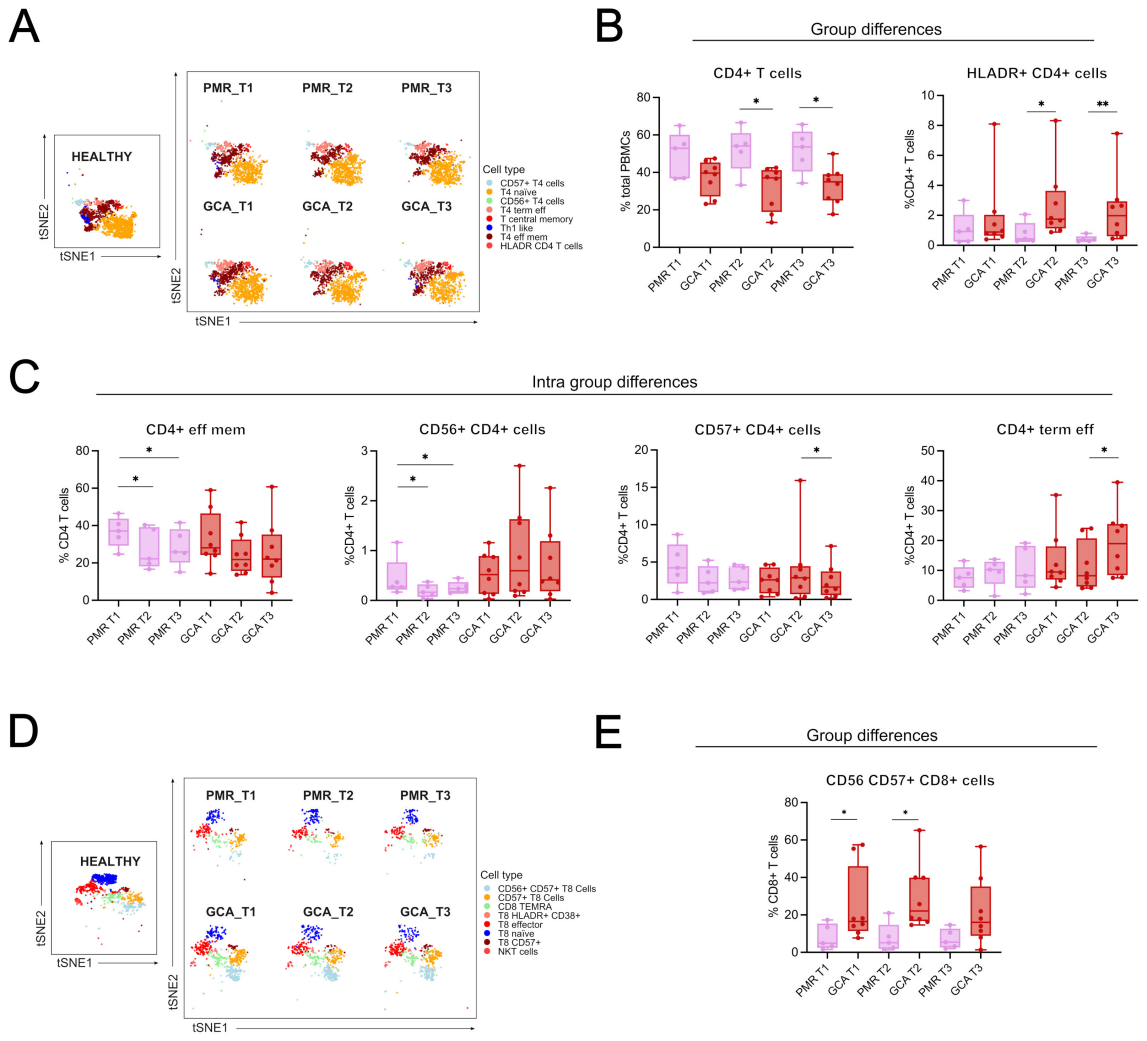
Group and intra-group differences in immune cell types/subsets across time. **(A)** t-distributed stochastic neighbor embedding (t-SNE) plot visualizing the clustering of CD4^+^ T cell subphenotypes from healthy individuals, PMR, and GCA patients across three time points (T1: before GC treatment; T2 and T3: 48 and 96 hours after treatment, respectively), and a boxplot showing between-group differences in total CD4^+^ T cell frequencies. **(B)** Boxplots showing differences between GCA and PMR patients in total CD4^+^ T cells and HLADR^+^ CD4^+^ T cells. **(C)** Intra-group differences in PMR patients include a reduction in CD4^+^ effector memory T cells (T1 vs. T2 and T1 vs. T3), CD56^+^ CD4^+^ T cell frequencies (T1 vs. T2 and T1 vs. T3), and CD4^+^ terminal effector T cells increased from T2 to T3. Intra-group differences in GCA patients include a decrease in CD57^+^ CD4^+^ T cells (T2 vs T3) and an increase in CD4^+^ terminal effector T cells. **(D)** t-SNE plot showing clustering of CD8^+^ T cell subphenotypes across all groups and time points. **(E)** Boxplots showing intra-group differences in CD56^+^CD57^+^ CD8^+^ T cells, which were significantly increased in GCA patients compared to PMR patients at T1 and T2. Data are presented as box plots with dots showing individual patient measurements. The first quartile and the third quartile are shown in the lower and upper horizontal lines, respectively. The horizontal line in the boxes represents the median value. Between-group comparisons at each time point were assessed using the Kruskal–Wallis test, followed by post hoc Mann–Whitney U tests with Bonferroni correction for GCA, PMR, and healthy individual samples. In the present figure, only GCA and PMR data are presented to give emphasis on the two disease groups. Intra-group comparisons (T1 vs T2 and T2 vs T3) were assessed using the Friedman test for one-way repeated analysis. *p < 0.05, **p < 0.01. CD, Cluster of Differentiation; T4, CD4+ T cells; T8, CD8+ T cells.

Group comparisons were made to identify differences between GCA and PMR patients (**Figures 2A–E**). Two subsets were increased in GCA patients compared to PMR patients. HLADR+ CD4+ T cells were found increased at T2 and T3 (**Figures 2A, B**), and CD56+CD57+ CD8+ T cells were also found increased in T1 and T2 (**Figures 2D, E**). No further differences in immune cell subtypes were found between the two disease groups.

Intra-group comparisons were made to depict the changes in immune cell subtypes/subphenotypes over time. Differences in the kinetics of only four CD4+ T cell subsets were observed. CD4+ effector memory T cell and CD56+ CD4+ T cell (NKT-like) frequencies were decreased from T1 to T3 in the PMR group. CD57+ CD4+ T cell frequencies were decreased, while CD4+ terminal effector cell frequencies were increased from T2 to T3 in the GCA group (**Figures 2A, C**). The kinetic differences observed may indicate that GC can affect these cell types in the first 96h of treatment. No further differences were observed in the kinetics of other immune cell subtypes/subphenotypes in GCA and PMR patients during the first 96 hours of treatment.

### 3.2 Early effects of steroids in distinct immune cell subsets in PMR and GCA patients

**Figure 3:**
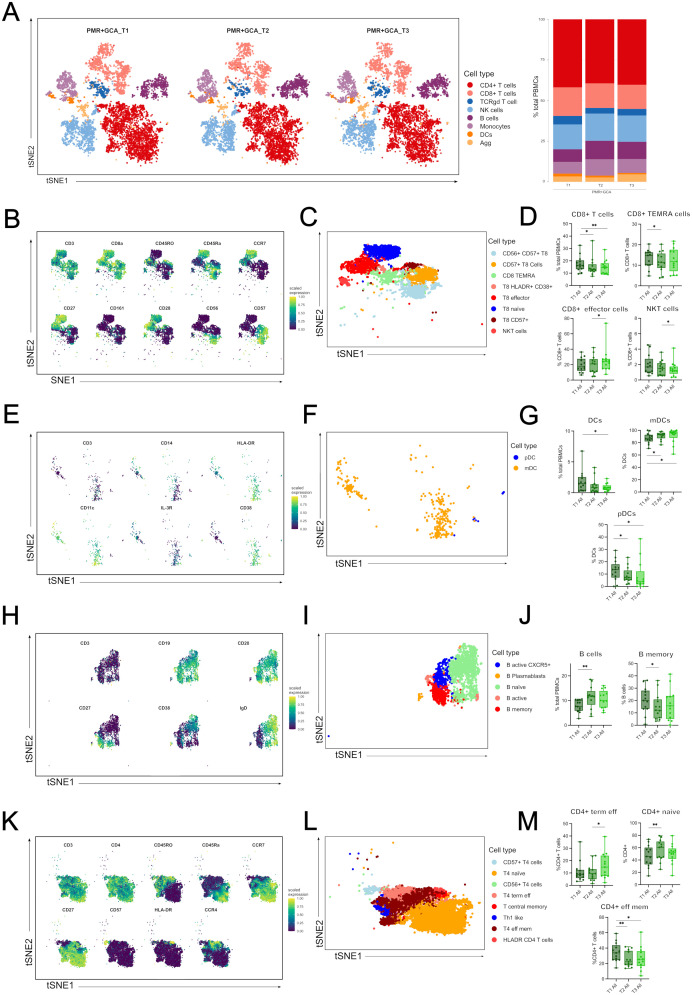
Immune cell types and sub-phenotypes kinetics in the first 96 hours of GC treatment in GCA and PMR patients. **(A)** A t-distributed stochastic neighbor embedding (t-SNE) analysis was performed, and a plot was generated visualizing cluster assignments of main cell types in all patients (PMR and GCA) before steroid administration (T1) (n=14), at 48 hours (T2) (n=14) and 96 hours (T3) (n=13) after steroid administration (left panel). The composition of the major immune populations within total PBMCs (CD45+ immune cells) at each time point (T1-T3) in PMR, and GCA patient samples combined, is represented by vertical bars; the length of the colored segment represents the proportion of cells as a percentage of PBMCs. Colors represent different populations within PBMCs **(B)** A t-SNE plot visualizes the mean expression of main markers characterizing CD8+ T cells and CD8+ sub-phenotypes (blue: no or low expression; green: high expression), **(C)** A t-SNE plot visualizing the sub-phenotypes of CD8+ T cells, **(D)** Box plots of total CD8+ cells and CD8+ sub-phenotypes (CD8+ TEMRA cells, CD8+ effector memory T cells, and NKT cells), **(E)** A t-SNE plot visualizes the main markers’ mean marker expression used to characterize DCs and DCs sub-phenotypes (blue: no or low expression; green: high expression), **(F)** A t-SNE plot visualizing the sub-phenotypes of DCs, **(G)** Box plots of total DCs and DC subsets (mDCs, and pDCs). **(H)** A t-SNE plot visualizes the main markers’ mean marker expression used to characterize B cells and B cell sub-phenotypes (blue: no or low expression; green: high expression), **(I)** A t-SNE plot visualizing the subphenotypes of B cells, **(J)** Box plots of total B cells and B memory cells. **(K)** A t-SNE plot visualizes the mean expression of main markers characterizing CD4+ T cells and CD4+ sub-phenotypes (blue: no or low expression; green: high expression), **(L)** A t-SNE plot visualizing the sub-phenotypes of CD4+ T cells, **(M)** Box plots of CD4+ phenotypes (CD4+ terminally effector memory T cells and CD4+ effector memory T cells) frequencies calculated as the percentage of total CD4+ T cells. Only statistically significant data are presented. Data are presented as box plots with dots showing individual patient measurements. P-values were determined by Wilcoxon t-test for paired analysis. *P<0.05, **P<0.01. The first quartile and the third quartile are shown in the lower and upper horizontal lines, respectively. The horizontal line in the boxes represents the median value. TEMRA, terminally differentiated effector memory cells; NKT, Natural Killer T-like cells; DCs, dendritic cells; mDCs, myeloid DCs; pDCs, plasmacytoid DCs; CD, cluster of differentiation; CCR7, C-C chemokine receptor type 7; IL-3R, interleukin-3 receptor (CD123); T4, CD4+ T cells; T8, CD8+ T cells.

Next, we investigated the kinetics of major immune cell compartments and specific cell sub-phenotypes by pooling all patients irrespective of their diagnosis to identify those cell types affected by GC administration during the acute inflammatory response (**Figure 3A**). The median frequencies of each immune cell type and sub-phenotypes detected in all patients and healthy individuals’ PBMC samples are presented in **Supplementary Table 3**. Among the major immune cell types, total CD8+ T cells were decreased after 48h (T2) and 96h (T3) of GC administration (T3) (**Figures 3B–D**). Total DCs were also decreased after 96h (T3) of GC initiation (**Figures 3E–G**). On the contrary, total B cells were increased shortly after GC administration (48h) (**Figures 3H–J**).

Further analysis revealed differential frequencies within each major immune cell subset. In the CD8+ T cell subgroup, CD8+ TEMRA cells were decreased at T2 vs T1, CD8+ effector T cells were increased at T3 vs T2, and CD56+ CD8+ T cells (NKT-like) were decreased at T3 vs T1 (**Figure 3D**). Myeloid dendritic cells (mDCs) displayed an increased frequency at T2 vs T1 and T3 vs T1, while plasmacytoid DCs (pDCs) were decreased at T2 vs T1 and T3 vs T1 (**Figure 3G**). In the B cell subgroup, only B memory cells decreased at T2 vs T3 (**Figure 3J**). Although CD4+ T cells did not show any kinetic alterations, differences were observed in CD4+ T cell subsets. CD4+ naïve T cells were increased at T2 vs T1, CD4+ terminal effector T cells were increased at T3 vs T1, and CD4+ effector memory T cells were decreased at T2 vs T1 and T3 vs T1 (**Figures 3K–M**).

### 3.3 Cytokines and chemokines are not sensitive-to-change molecules in the early phases of steroid treatment

**Figure 4:**
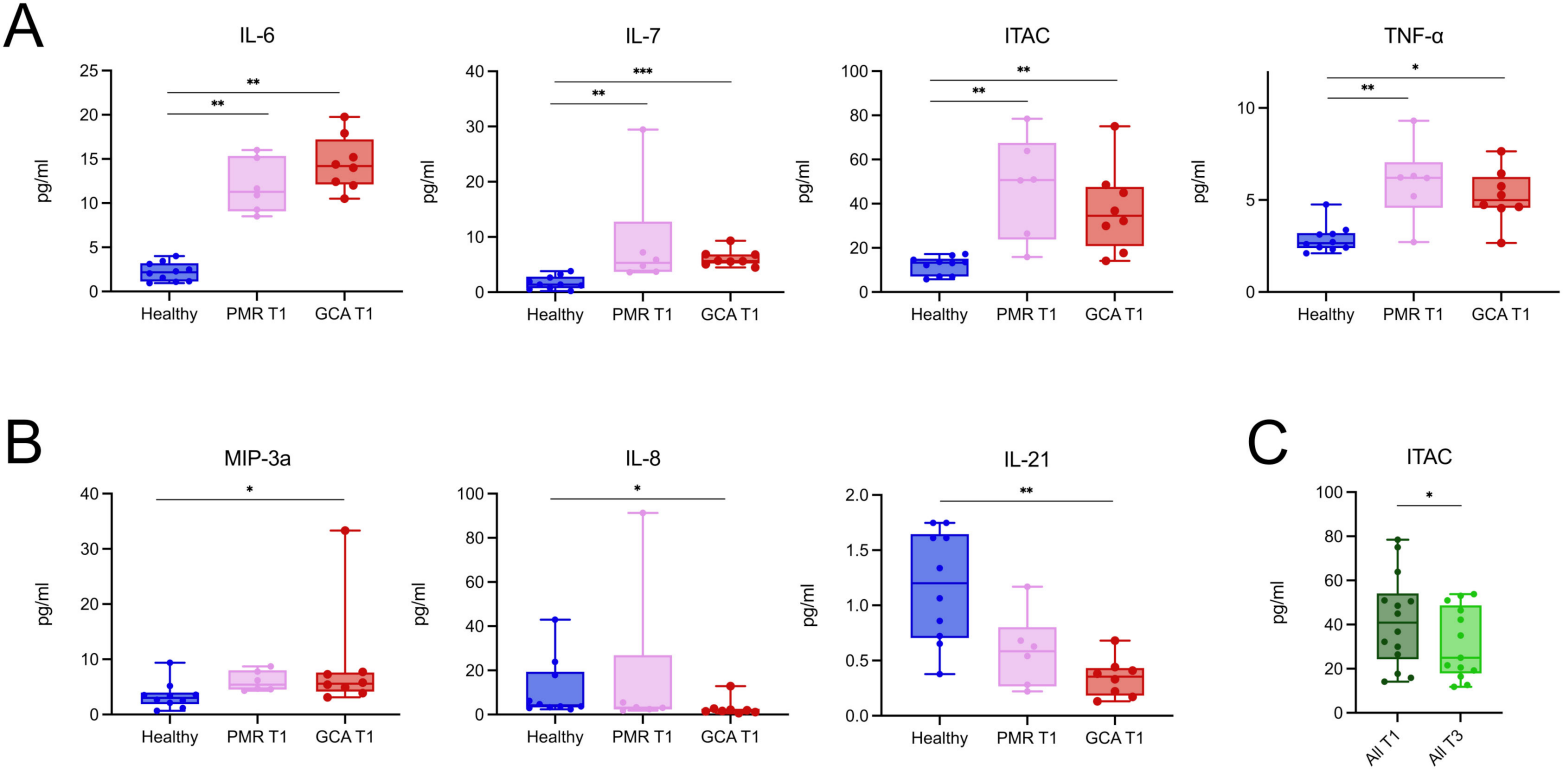
PMR and GCA patients exhibit a relatively similar and stable serum cytokine profile during the early phase of glucocorticoid treatment. **(A)** Serum concentrations of IL-6, IL-7, ITAC, and TNF-α were significantly elevated in both PMR (n = 6) and GCA (n = 8) patients at diagnosis (T1) compared to healthy individuals (n = 10). **(B)** MIP-3a was selectively increased in the GCA group, while IL-8 and IL-21 were decreased in GCA patients compared to healthy controls. **(C)** When PMR and GCA patients were pooled, only ITAC levels showed a statistically significant decrease after 96 hours of steroid treatment (T3), indicating that the overall serum cytokine profile remained relatively stable during this early treatment phase. Data are presented as box plots with dots showing individual patient measurements. The first quartile and the third quartile are shown in the lower and upper horizontal lines, respectively. The horizontal line in the boxes represents the median value. Comparisons in panels A and B were performed using the Kruskal–Wallis test followed by Dunn’s *post hoc* test with Bonferroni correction. Comparisons in panel C were performed using the Wilcoxon matched-pairs signed-rank test.*p < 0.05, **p < 0.01, **p < 0.001. IL, Interleukin; TNF-α, Tumor Necrosis Factor-alpha; ITAC, Interferon-inducible T cell alpha chemoattractant; MIP-3a, Macrophage Inflammatory Protein-3 alpha; T1, time of diagnosis; T3, 96 hours after glucocorticoid treatment initiation. *p < 0.05, **p < 0.01, ***p < 0.001.

To study the cytokine profile, we tested a Luminex panel of 21 cytokines and chemokines against the serum of patients and healthy individuals. Firstly, we sought to identify differences in the serum cytokine profile between groups in T1, at diagnosis. IL-6, IL-7, ITAC, and TNF-α levels were increased in the GCA and PMR groups vs the healthy individual group (**Figure 4A**). MIP-3a levels were increased in the GCA group, while IL-8 and IL-21 levels were decreased in the GCA group (**Figure 4B**). No further statistically significant differences were observed in the levels of the cytokines and chemokines tested during diagnosis (**Supplementary Table 4**).

To study the effect of GC on serum cytokine profile, we pooled all patients irrespective of their diagnosis to identify cytokines and chemokines sensitive to change in the first 96 h upon GC treatment. Surprisingly, only ITAC levels were decreased after 96 h of steroid treatment (**Figure 4C**). In contrast, the levels of the rest of the cytokines and chemokines remained stable between time points (**Supplementary Figure 3**). These findings suggest that, in the early phases of GC treatment, cytokines and chemokines are generally not sensitive to change, with the notable exception of ITAC.

### 3.4 Overall plasma levels of pro-inflammatory lipid mediators remain increased even after steroid treatment in GCA patients

**Figure 5:**
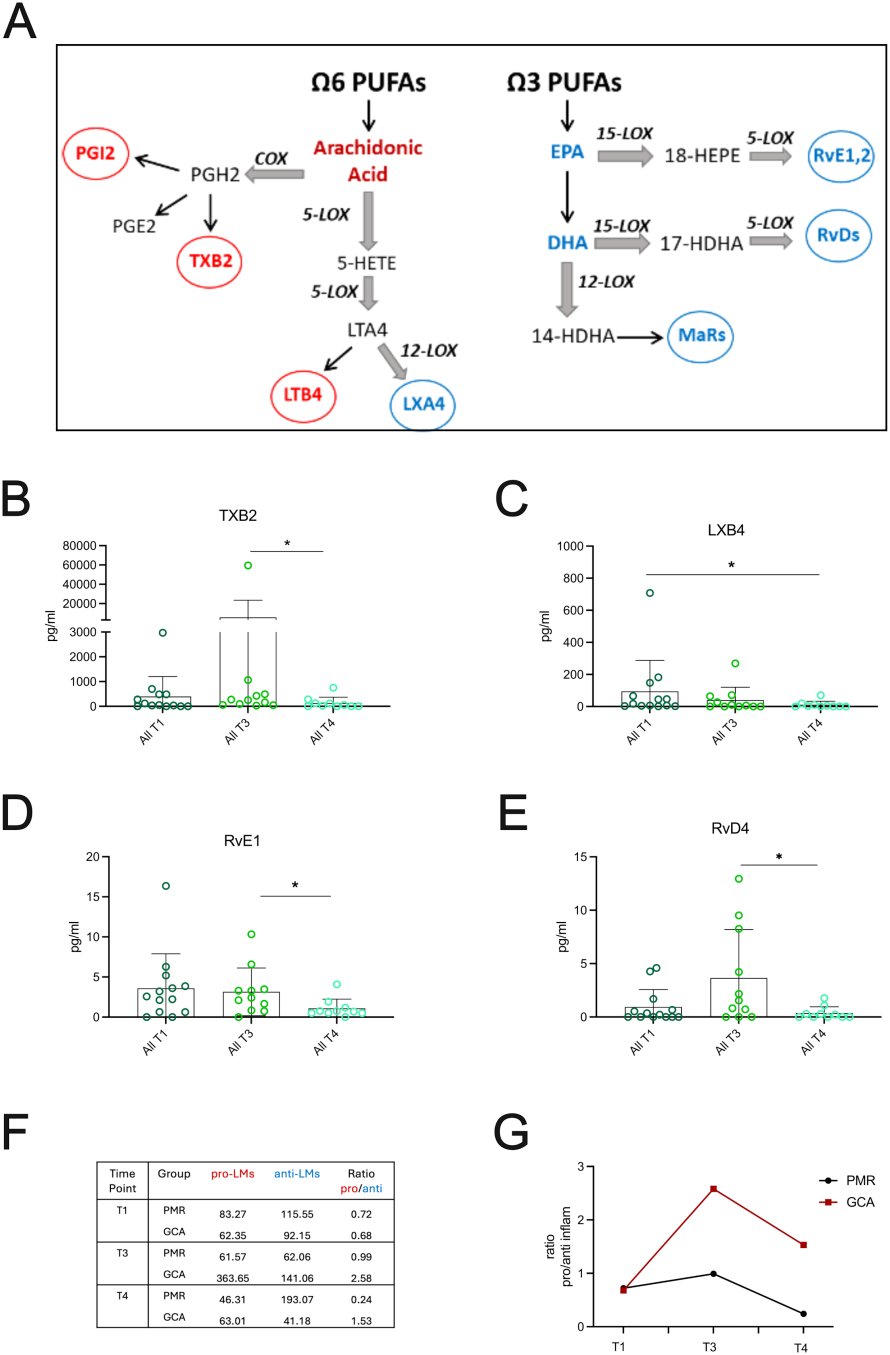
GC treatment effects on Lipid Mediators in GCA and PMR patients. **(A)** Schematic representing the major ω6 and ω3 derived metabolites measured in patient cohort. **(B–E)** Plasma levels of the most important lipid mediators (LMs) derived from AA, EPA and DHA metabolism from PMR (n=6) and GCA (n=7) patients (total, n=13) during active phase (T1), after 4days (T3) and 6 months after GC treatment (T4). Data are presented as box plots with dots showing individual patient measurements. P-values were determined by Mann Whitney t-test for non-parametric comparisons. *P<0.05. **(F)** Representative table of the average of pro-inflammatory LMs (median values of PGE2, 6k-PGF1a, TXB2 and LTB4) to anti-inflammatory SPMs (median values of LXA4, LXB4, Mar2, PDX, PD1, RvD1-5, RvE1,2,4) and **(G)** The average of the median of the well-characterized pro-inflammatory LMs: PGE2, 6k-PGF1a, TXB2 and LTB4 to the average of the median of well-known small lipid mediators (SPMs): LXA4, LXB4, Mar2, PDX, PD1, RvD1–5 series and RvE1, RvE2, RvE4 series was plotted per patient group. The ratio of pro/anti-inflammatory LMs is increased in GCA vs PMR patients after 96 hours of steroid treatment (T3) and is still increased after 6 months of steroid treatment (T4). COX, Cyclooxygenase; EPA, Eicosapentaenoic Acid; DHA, Docosahexaenoic acid; GC, glucocorticoids; HDHA, hydroxy Docosahexaenoic Acid; HEPE, hydroxy-eicosapentaenoic acid; HETE, Hydroxy-eicosatetraenoic acid; LOX, Lipoxygenase; LTA4, Leukotriene A4; LXA4, Lipoxin A4; LXB4, Lipoxin B4; MaRs, Marezins; PGE2, Prostaglandin E2; PGH2, Prostaglandin H2; PGI2, Prostaglandin I2; PUFA, Polyunsaturated Fatty Acid; Rv, Resolvin; TXB2, Thromboxane B2.

To study LMs levels in GCA and PMR patients during the active phase of the disease (T1), and the effect of short (4 days, T3) and prolonged (6 months, T4) GC treatment, we performed targeted lipidomics for a total of 25 LMs, covering the most known LM metabolites (**Figure 5A**). Initially, plasma levels at T1, T3, and T4 time points were evaluated. Among the 25 bioactive LMs measured in GCA and PMR patients, from arachidonic acid metabolism, only thromboxane B2 (TXB2) and lipoxin B4 (LXB4) were statistically altered by GC treatment, as shown in **Figures 5B, C**. The concentration of TXB2, a well-known pro-inflammatory and pro-thrombotic LM, showed a tendency to increase after 4 days of GC administration (T3) (**Figure 5B**), indicating that treatment did not affect its levels. Prolonged GC treatment (T4), resulted in a significant reduction of TXB2 levels (**Figure 5B**), suggesting that the reduction of TXB2 levels may require high cumulative GC dose. In contrast, LXB4, a member of lipoxins, which are the first SPMs derived from arachidonic acid (AA) metabolism, showed an increase during the active phase and displayed a significant decrease following prolonged GC treatment (**Figure 5C**), implying that GC treatment is also able to reduce LXB4 levels.

Regarding ω3 PUFAs metabolism, RvE1, a key SPM derived from eicosapentaenoic acid (EPA) metabolism, was increased during the active phase (T1), with its levels dropping only after prolonged GC treatment (T4) (**Figure 5D**). Another important pro-resolving mediator, RvD4, a well-known docosahexaenoic acid (DHA)-derived SPM, was increased after 96 hours of GC treatment (T3) and was significantly decreased after prolonged GC treatment (T4) (**Figure 5E**). LMs and SPMs that didn’t show any statistically significant changes are presented in **Supplementary Figure 4**. Collectively, 6 months of GC administration significantly lowered distinct pro-inflammatory LMs and SPMs levels in the plasma of all patients tested.

When LMs’ plasma levels were compared between groups, GCA and PMR patients did not show any statistical significance regarding specific LM or SPM (data not shown). However, when the ratio of pro-inflammatory LMs to pro-resolving LMs (SPMs) was evaluated at different time points (T1, T3, T4) for PMR and GCA patients (**Figures 5F, G**), it was found that GCA patients were unable to resolve their inflammatory milieu after a short period of GC treatment. Interestingly, the ratio remained high even after a long period of GC treatment (**Figure 5G**), suggesting that pro-inflammatory LMs might remain active in the periphery.

### 3.5 Dynamic remodeling of lipid mediators–immune subsets correlations across steroid treatment time

**Figure 6:**
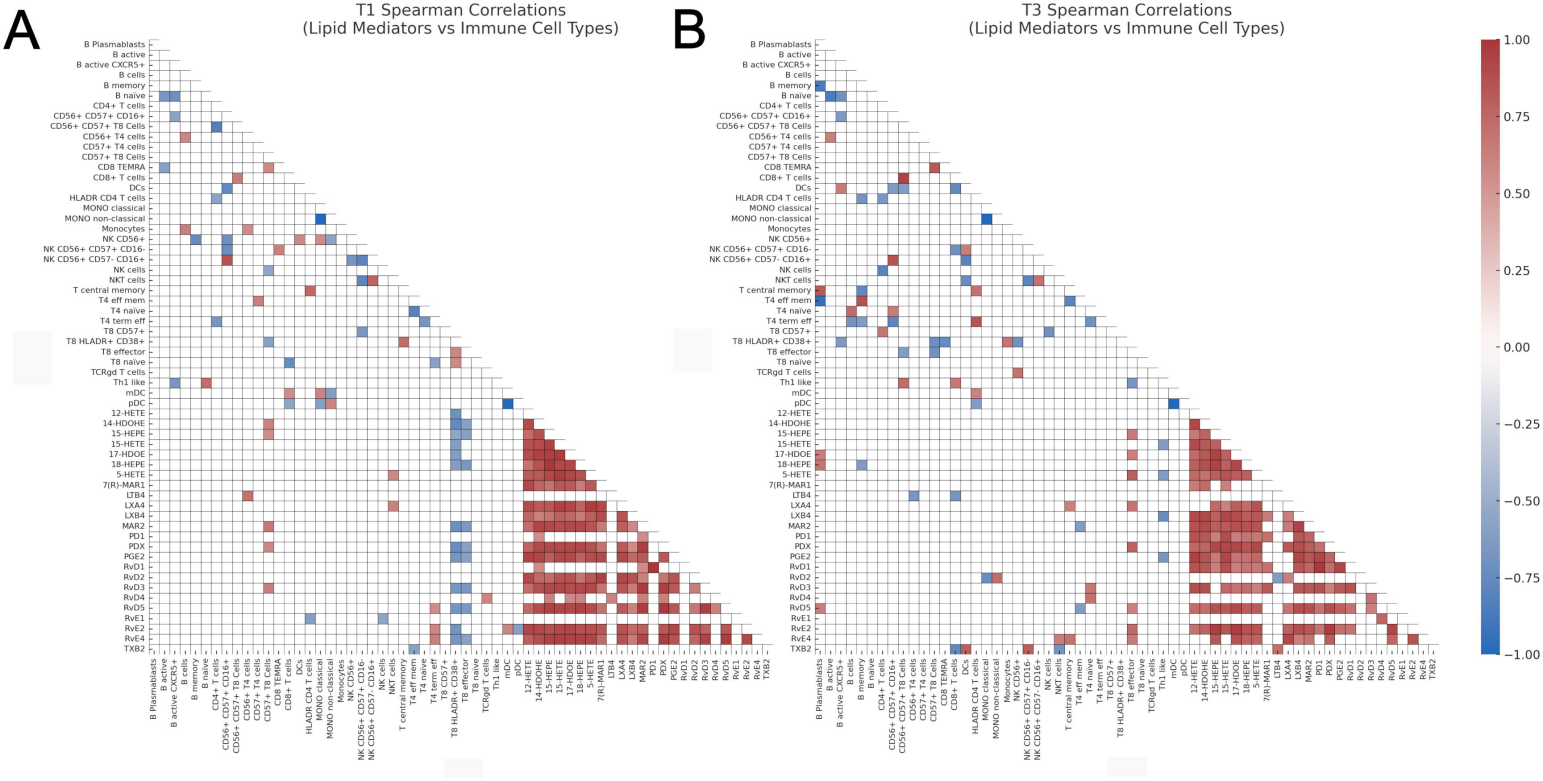
Heatmaps of Spearman correlation coefficients between lipid mediators and immune cell types. **(A)** Correlation analysis at T1, and **(B)** Correlation analysis at T3. Only statistically significant correlations (p < 0.05, two-tailed Spearman test) are shown. Correlations were computed independently for each time point. The color scale represents the Spearman r coefficient (range: -1 to 1), and only the lower triangle of the correlation matrix is displayed for visual clarity. CD, cluster of differentiation; DCs, dendritic cells; HEPE, hydroxy-eicosapentaenoic acid; HETE, hydroxy-eicosatetraenoic acid; IL-3R, interleukin-3 receptor (CD123); LTB4, leukotriene B4; PDX, Protectin DX; PD1, Protectin D1; 17-HDOE, 17(S)-hydroxydocosahexaenoic acid; LXA4, lipoxin A4; LXB4, lipoxin B4; MaR1, maresin 1; mDCs, myeloid dendritic cells; NK, natural killer; NKT, natural killer T-like cells; pDCs, plasmacytoid dendritic cells; PGD2, prostaglandin D2; PGE2, prostaglandin E2; Rv, resolvin; TEMRA, terminally differentiated effector memory cells; TXB2, thromboxane B2; T4, CD4+ T cells; T8, CD8+ T cells.

When focusing on the correlations between lipid mediators and immune cell types, several dynamic patterns emerged across T1 and T3. Some correlations observed at T1 were reversed in the direction at T3 (**Figure 6**). These included 15-HEPE vs CD8+ effector (T1: r = -0.60, p = 0.0340; T3: r = 0.65, p = 0.0336), LTB4 vs CD56+ CD4+ T cells (T1: r = 0.70, p = 0.0098; T3: r = -0.66, p = 0.0303), and PDX vs CD8+ effector T cells (T1: r = -0.58, p = 0.0402; T3: r = 0.78, p = 0.0066), suggesting substantial shifts in the regulatory dynamics between these lipid mediators and immune subsets.

In contrast, several significant correlations at T1 were no longer present at T3. These included 12-HETE vs CD8+ HLADR+ CD38+ T cells (T1: r = -0.71, p = 0.0086), 14-HDOHE vs CD57+ CD8+ T cells (T1: r = 0.61, p = 0.0302), 14-HDOHE vs CD8+ HLADR+ CD38+ T cells (T1: r = -0.75, p = 0.0042), 14-HDOHE vs CD8+ effector T cells (T1: r = -0.59, p = 0.0360), and 15-HEPE vs CD57+ CD8+ T cells (T1: r = 0.57, p = 0.0473), indicating a loss of association or regulatory engagement over the GC treatment time (**Figure 6A**).

Conversely, new correlations emerged at T3 that were not significant at T1. Notable examples include 15-HETE vs Th1 like cells (T3: r = -0.62, p = 0.0462), 17-HDOE vs B plasmablasts (T3: r = 0.64, p = 0.0402), and 17-HDOE vs CD8+ effector T cells (T3: r = 0.62, p = 0.0478) (**Figure 6B**). These findings suggest the emergence of novel lipid–immune cell interactions as the time increases after GC treatment initiation.

## 4 Discussion

In this brief report, we have prospectively evaluated the acute phase responses in patients with GCA and/or PMR using high-end technologies to detect changes in the main peripheral blood immune cell populations and subpopulations, serum cytokines/chemokines, and small lipid mediators. Our main purpose was to get further insights into steroid action in the acute systemic inflammatory response, during the early phases of treatment, to identify new biomarkers with a strong pathogenetic relevance, that could be used as treatment selection or monitoring tools. We identified alterations in major immune cell populations, with a reduction of CD4+ T cells associated exclusively with the GCA group. The kinetic study revealed a reduction in CD8+ T cells and DCs and an increase in B cell numbers in both GCA and PMR patients, across the different time-points. Deep phenotyping disclosed that these changes were associated with alterations in the immune subsets constituting these major immune cell populations. Surprisingly, the only chemokine that was altered in the first 4 days of treatment was ITAC. Lipidomic analysis revealed a persistent pro-inflammatory LM profile after short-term GC treatment that was still evident after 6 months in the treatment course exclusively in the GCA group.

Even though the pathogenetic mechanisms of GCA have not been elucidated, it appears that T cells play a central role. Indeed, increased numbers of CD4+ T cells with a polarization toward Th1 and Th17 cells have been described in tissue lesions (18). The persistent reduction of CD4+ T cells in the peripheral blood of GCA, but not in PMR patients reported in the present study could be attributed to the migration and localization within the inflamed tissues (2). The same mechanism could also explain the reduced peripheral blood frequencies of CD8+ T cells and DCs, as both are increased in tissue lesions during active disease (19, 20).

One of the major aims of the present study was to decipher the role of the early effect of GC in peripheral immune cell populations in both GCA and PMR. Glucocorticoids exert broad immunomodulatory effects on peripheral blood cells, including the suppression of pro-inflammatory cytokine production and modulation of leukocyte distribution. In general, GC induce lymphocyte apoptosis (especially T cells), decrease monocyte activation and antigen presentation, reduce eosinophil and basophil counts, and cause transient neutrophilia (21–23). These effects are mediated via the glucocorticoid receptor (GR), which upon activation, translocates to the nucleus and either represses transcriptional factors like NF-κB and AP-1 or promotes anti-inflammatory gene expression such as lipocortin-1 (annexin A1) (24). The identification of cell alterations at the initiation of treatment will potentially offer not only new insights into the mechanism of acute inflammatory response, but also new cellular biomarkers, relevant to pathogenetic mechanisms, and if further validated, new targets of treatment. One of the most prominent findings in this report is the reduction of CD8+ T cells, after 48 hours post-treatment, affecting mainly terminally differentiated CD8+ T cells (TEMRA) and NKT-like T cells that are observed in other chronic inflammatory conditions (25, 26). The effect of steroids in temporal artery biopsies (TABs) has been studied in TABs obtained at different time points after GC treatment initiation. DCs and CD4+ T cells are decreased in the inflamed tissue after treatment initiation, probably due to the apoptotic effects of GC (20). CD8+ T cells are present in the temporal artery and aorta biopsies of GCA patients. Peripheral blood CD8+ T cells have been reported to be decreased during the active phase of the disease (19), while an increase in distinct cytotoxic CD8+ T cell subsets (perforin+ Granzyme B+) has been identified in the peripheral blood of active GCA patients (27). Taken together, our data along with other literature reports indicate that CD8+ T cells appear to be involved in the inflammatory operating mechanisms of GCA, with GC treatment being effective in reducing their numbers even after 48h of treatment initiation. We speculate that this reduction is probably attributed to the apoptotic effect of GC in this cell type, similar to that observed in CD4+ T cells, but this should be verified by further experiments.

In an attempt to differentiate GCA from PMR, we found a difference in CD56+ CD57+ CD8+ T cells, which were increased in the peripheral blood of GCA patients, as compared to PMR patients. These cells possess a highly cytotoxic phenotype after *in vitro* stimulation, characterized by aberrant IFN-γ and IL-13 production (28). CD56+ CD8+ T cells are decreased after 96 hours of steroid treatment in the current study, suggesting that steroids target early and effectively this cell population with high inflammatory properties. The cell surface expression of CD57 on CD8+ T cells has been linked to exhausted CD8+ T cells that possess a replicative senescence phenotype with upregulated IFN-γ production (29, 30). This immune subset underpins a highly cytotoxic CD8+ subset with decreased proliferative capacity, and features of replicative senescence and T-cell exhaustion (31). Since these CD8+ T cell sub-phenotypes haven’t been studied in GCA before, the investigation of their transcriptome and function both in the peripheral blood and inflamed tissue may offer further insights into their role in GCA’s pathogenetic mechanism

In the present study, there were no significant changes in the kinetics of serum cytokines and chemokines tested. Christ et al. reported the kinetics of the serum proteome of GCA patients at 0, 3, and 10 days and 1, 6, and 12 months after tocilizumab treatment (11). The authors reported no significant changes in the first 3 days of GC initiation treatment in serum cytokines but observed significant changes after 10 days of tocilizumab monotherapy. Collectively, these results indicate that cytokine and chemokine inflammatory mediators in the blood are not related to the abrupt change in the clinical picture of GCA and PMR patients. However, we report that ITAC levels are reduced in the serum of patients after 4 days of GC treatment, a finding that wasn’t validated in the study of Christ et al. (11). ITAC (CXCL11) is a chemokine and a strong ligand of the chemokine receptor CXCR3 expressed on the surface of multiple cells, including activated T cells. The CXCL11-CXCR3 axis is associated with Th1 responses (32) and maintenance of T cell infiltrates, as shown in other inflammatory diseases (33). Thus, the reduction of ITAC levels after 96 hours of treatment with GC may be associated with abrogated Th1 responses following the abrupt change in the clinical picture of GCA and PMR patients after 48–96 hours of GC treatment.

Previous work from our laboratory, implementing metabolomics analysis in GCA and PMR serum, has identified a deregulated lipid metabolism profile in these patients before and after steroid treatment (34). For this reason, in the current study, lipidomics analysis was implemented, focusing on lipid mediators, to better understand the effector mechanisms taking place in GCA and identify potential biomarkers for improved early diagnosis, classification, and treatment. Although LMs have a crucial role in regulating inflammation, they have never been studied in the context of GCA pathogenesis. Our results revealed that the ratio of pro-inflammatory to pro-resolving LMs in GCA patients remains increased, despite the higher GC dose and the reduction in inflammatory markers after 6 months of GC treatment. In particular, at T4, pro-inflammatory LMs reach the levels detected at T1, while pro-resolving LMs, which play an anti-inflammatory role, are decreased compared to T1. These findings suggest either that LM production can be regulated by GC or that impaired clearance may occur, both resulting in tissue damage and inappropriate tissue remodeling.

As shown in previous studies, arachidonic acid metabolism may play a key role in aneurysm formation as seen in Marfan’s Disease (35) and steroid-induced aneurysm formation in mouse models (36), highlighting the role of ω6 PUFA metabolism in tissue remodeling. A possible explanation behind the impaired clearance of pro-inflammatory LMs could be due to defects in their key metabolic enzymes, such as lipoxygenases (LOX), cyclooxygenases (COX), or cytochrome P450 enzymes (37), or enzymes responsible for their excretion from urine (i.e., UGTs) (38). Our study found that LM thromboxane B2 (TXB2) levels- a key vasoactive LM- were low during the acute phase and increased after 4 days of GC treatment. Under normal conditions, it is completely cleared in the urine (39). During the active phase of the disease, TXB2 levels are expected to be elevated due to excessive inflammation, unless patients are under antiplatelet drugs. However, few of our patients are under antiplatelet treatment, suggesting that GC are not able to affect TXB2 levels directly or indirectly. On the other hand, pro-resolving LMs (SPMs) with known anti-inflammatory effects (40), such as lipoxin B4 (LXB4) and resolvins E1 (RvE1), were increased during the active disease phase (T1, T3), following reports in other inflammatory conditions (8, 40), probably as an endogenous mechanism to counteract inflammation. Short-term GC treatment was not able to increase their levels, as described in mouse models of airway inflammation (41), but also in GC-treated human monocytes (42). In contrast in our study, resolvin D4 (RvD4) levels increased after 4 days of GC treatment, indicating that steroids may selectively affect the production of specific SPMs. However, RvD4 levels dramatically decreased after long-term GC treatment, suggesting a dose-dependent effect of GC on SPM levels.

It is known that certain drugs can influence the production of LMs and SPMs (43). GC treatment has long been known to affect LM production, but its main effect is the reduction of pro-inflammatory eicosanoids by interfering with inducible COX-2 and cPLA2α (44). Several studies indicate that corticosteroids not only limit inflammation but also promote the resolution phase. However, the cellular and molecular basis of GC's actions underlying the resolution of inflammation is partially known (41, 42).

An explanation beyond the context of GCA-specific inflammation, covering the metabolic effects of GC, notes that GC can increase lipolysis (45), particularly after 48h of exposure (46). This means that increased lipolysis will result in more free fatty acids, including ω6 and ω3 PUFAs, which are pro-inflammatory and pro-resolving (SPMs) LMs precursors. We hypothesize that the pro-inflammatory ratio may remain elevated in GCA patients due to impaired enzymatic function. It is known that high doses of GC may inhibit the biosynthesis of some SPMs by blocking key enzymes (i.e 5-LOX and 15-LOX) (47, 48), and their inhibitory actions of RvD1 and 17-HDHA on antibody production in B cells (49). Given that these enzymes are present in many different cell types (immune cells, epithelial cells, hepatocytes, adipocytes) (8) and that the metabolic effects of GC are widespread across multiple systems (cardiovascular, musculoskeletal, and immune system) (50), there may be a significant association between these enzymes and a potential explanation for the relationship between GC metabolic effects and LM production.

Overall, we hypothesize that a defect in SPM biosynthesis pathways promoting residual tissue inflammation is taking place in a portion of GCA patients. These patients are prone to disease relapse, presenting also with aneurysm formation in the latter years following disease diagnosis. The action of steroids in key biosynthetic enzymes (i.e., LOXs) operating in human monocyte/macrophage populations (42) remains to be investigated in GCA. We strongly believe that further research on these pathways may pave the way to new treatment targets and/or biomarkers to predict disease comorbidities such as the formation of thoracic aortic aneurysms (2, 51), and it is currently a working project of our group.

To our knowledge, this is the first time in the literature that the early immunological changes, shortly after steroid treatment initiation, are being investigated. The strengths of this study include the employment of different immunological data derived from high-end technologies analysis (Luminex Assay, CyTOF analysis, and lipidomics analysis), prospectively, at three time points early during the disease course. All methods have been performed employing a single sampling of patients. Our study highlights specific immune cell subsets that are differentially altered during the early phases of steroid treatment, that require further research with functional experiments and an altered proinflammatory lipid mediator profile still evident after 6 months of treatment, indicating new therapeutic pathways of research in the underlying tissue inflammation. The limitations of our study include the small number of patients and the single-center nature of the study. There is also an inherent difference in the GC treatment strategy between GCA and PMR that may have affected the results of our study. The data produced in the present study, coupled with transcriptomic data on peripheral blood immune cells and the investigation of tissue immunophenotype by spatial proteomic analysis, would offer a comprehensive and in-depth analysis of the immune cell repertoire in the early inflammatory cascade recapitulating the underlying pathophysiology of GCA. Moreover, in the present report, we haven’t studied the impact of steroids on healthy individuals. It would be interesting to investigate the immune cell type frequencies and lipidome of healthy individuals receiving GC for a short period, and this is an unmet need to be addressed in future studies. Finally, CyTOF analysis of granulocytes and data about immune cell subpopulation frequencies after 6 months of steroid treatment is not included in the present study, which would better characterize immune cell type repertoire during deep remission.

Nevertheless, the results described above, hold the promise of new biomarker discovery if the patient population is larger, and offer the possibility of integrative analyses, to better understand the mechanism of chronic inflammation in GCA/PMR.

## 5 Conclusions

Our preliminary results identified for the first time distinct immunological changes in the peripheral blood of GCA and PMR patients associated with the rapid response to steroid treatment. By encompassing data at 3-time points during the early phases of steroid treatment, we highlight kinetic changes in CD4+ T cells, CD8+ T cells, and DCs and subpopulations/sub-phenotypes of these major immune cell types. Furthermore, ITAC/CXCL11 serum levels are also altered upon steroid initiation treatment and combined with kinetic changes in CD4+ T cells, we hypothesize that the CXCR3-CXCL11 axis may play a role in the rapid clinical improvement of these patients. These findings require further investigation by incorporating the transcriptomic profile along with functional studies on these cell types to decipher their role in the resolution of inflammation. Intriguingly, distinct lipidomic profiles of GCA and PMR patients could discriminate the two patient groups after 6 months of treatment, with GCA patients showing a pro-inflammatory LMs profile that requires further research to determine whether pro-resolving lipid mediator pathways are deregulated in these patients or the profile identified is an adverse effect of steroid treatment. Future studies could benefit patients and may provide new drug targets to restore LMs pathways and promote the resolution of the inflammatory response.

## Data Availability

The original contributions presented in the study are included in the article/**Supplementary Material**. Further inquiries can be directed to the corresponding author.
