# Supplementary material for: Multiparametric analysis in the peripheral blood of Giant Cell Arteritis and Polymyalgia Rheumatica patients at the early phases of steroid treatment reveals changes in cell subpopulations and lipid mediators: a preliminary study
Source: Front Immunol. 2025 Jun 4;16:1594263. doi: 10.3389/fimmu.2025.1594263 (PMC12175175; doi:10.3389/fimmu.2025.1594263)
Supplement: Supplementary file 1 [file Supplementaryfile1.docx]

Supplementary Material

Multiparametric analysis in the peripheral blood of Giant Cell Arteritis and Polymyalgia Rheumatica patients at the early phases of steroid treatment reveals changes in cell subpopulations and lipid mediators. A preliminary study

**Dimitris Anastasios Palamidas^1^, Maria Papadaki^2^, Nikolaos Paschalidis^3^, Eleftherios Pavlos^2,4^, Loukas Chatzis^1,2,5^, Ourania D Argyropoulou^1^, Panagiota Palla^1^, Dionysios Prevezanos^6^, Marc Dubourdeau^7^, Konstantinos Kambas^8^, Ioanna Evdokia Galani^2^, Andreas V Goules^1,5^, Evangelos Andreakos^2^, Athanasios G Tzioufas^1,2,5^**

^1^Department of Pathophysiology, School of Medicine, National and Kapodistrian University of Athens, Athens, Greece.

^2^Laboratory of Immunobiology, Center for Clinical, Experimental Surgery and Translational Research, Biomedical Research Foundation of the Academy of Athens, Greece.

^3^Mass Cytometry - CyTOF Laboratory, Center for Clinical Research, Experimental Surgery and Translational Research, Biomedical Research Foundation of the Academy of Athens (BRFAA), Athens, Greece

^4^Division of Basic Sciences, University of Crete Medical School, Heraklion, Greece.

^5^Research Institute for Systemic Autoimmune Diseases, Athens, Greece.

^6^Second Propedeutic Department of Surgery, National and Kapodistrian University of Athens, Athens, Greece.

^7^Ambiotis SAS, Toulouse, France

^8^Laboratory of Molecular Genetics, Department of Immunology, Hellenic Pasteur Institute, Athens, Greece.

*Correspondence:
Athanasios G Tzioufas
[agtzi@med.uoa.gr](mailto:agtzi@med.uoa.gr)

**Methods**

**Supplementary Table 1.** List of antibody-markers used for mass cytometry included in the MDIPA kit (Standard Biotools, California, USA).

|  | Target | Clone | Metal or fluorochromes | Localization |
| --- | --- | --- | --- | --- |
| 1 | CD45 | HI30 | 89 Y | Cell-surface |
| 2 | CCR6 | G034E3 | 141 Pr | Cell-surface |
| 3 | CD123 | 6H6 | 143 Nd | Cell-surface |
| 4 | CD19 | HIB19 | 144 Nd | Cell-surface |
| 5 | CD4 | RPA‐T4 | 145 Nd | Cell-surface |
| 6 | CD8a | RPA‐T8 | 146 Nd | Cell-surface |
| 7 | CD11c | Bu15 | 147 Sm | Cell-surface |
| 8 | CD16 | 3G8 | 148 Nd | Cell-surface |
| 9 | CD45RO | UCHL1 | 149 Sm | Cell-surface |
| 10 | CD45RA | HI100 | 150 Nd | Cell-surface |
| 11 | CD161 | HP‐3G10 | 151 Eu | Cell-surface |
| 12 | CCR4 | L291H4 | 152 Sm | Cell-surface |
| 13 | CD25 | BC96 | 153 Eu | Cell-surface |
| 14 | CD27 | O323 | 154 Sm | Cell-surface |
| 15 | CD57 | HCD57 | 155 Gd | Cell-surface |
| 16 | CXCR3 | G025H7 | 156 Gd | Cell-surface |
| 17 | CXCR5 | J252D4 | 158 Gd | Cell-surface |
| 18 | CD28 | CD28.2 | 160 Gd | Cell-surface |
| 19 | CD38 | HB‐7 | 161 Dy | Cell-surface |
| 20 | CD56 | NCAM16.2 | 163 Dy | Cell-surface |
| 21 | TCRgd | B1 | 164 Dy | Cell-surface |
| 22 | CD294 | BM16 | 166 Er | Cell-surface |
| 23 | CCR7 | G043H7 | 167 Er | Cell-surface |
| 24 | CD14 | 63D3 | 168 Er | Cell-surface |
| 25 | CD3 | UCHT1 | 170 Er | Cell-surface |
| 26 | CD20 | 2H7 | 171 Yb | Cell-surface |
| 27 | CD66b | G10F5 | 172 Yb | Cell-surface |
| 28 | HLADR | LN3 | 173 Yb | Cell-surface |
| 29 | IgD | IA6‐2 | 174 Yb | Cell-surface |
| 30 | CD127 | A019D5 | 176 Yb | Cell-surface |

**Results**

# CyTOF - Immune subsets annotation

The main immune cell types (CD4+ cells, CD8+ cells, TCRγδ cells, NK cells, DCs and Monocytes) were further divided into sub-phenotypes based on the cell surface marker expression pattern and assigned to the distinct phenotypes according to the literature. CD45RA, CD45RO, CD127, CCR4, CCR7, CD56 and CD57 marker expression pattern was used to annotate 8 clusters of CD4+ T cells as T4 naïve (Naïve; CD45RA+ , CD45RO- , CCR7+), T4 centr mem (Central Memory; CD45RA- , CD45RO+ , CCR7+ , CD27+ ), T4 eff mem (Effector Memory; CD45RA- , CD45RO+ , CCR7- , CD27+), T4 term eff (Terminal Effector; CD45RA- , CD45RO+ , CCR7- , CD27-), CD56+T4 (CD45RO+, CD127+, CD56+), CD57+T4 (CD45RO-, CD127+, CD57+), Th1-like T4 (CXCR5- , CCR4- , CXCR3+ , CCR6- , CD45Ra- , CD45RO+), Th2-like T4 (CXCR5- , CCR4+ , CXCR3+ , CCR6- , CD45Ra- ). Similarly, we annotated 8 subsets of T8 cells (CD3+ , CD8+ ) as T8 naïve (Naïve; CD45RA+ , CD45RO- , CCR7+), T8 TEMRA (T8 effector memory cells re-expressing CD45RA; CD45RA+), T8 effector memory (Effector Memory; CD45RA- , CD45RO+ , CCR7- , CD27+), T8 HLADR+ CD38+ (CD45RO+ , CD38+ , HLADR+ ), CD56+CD57+T8 Cells (CD45Ra+ ), CD57+T8 cells (CD45Ra+ ), CD27+CD28+CD57+T8 Cells (45RO+ , 45Ra-), and NKT (CD3+ , CD8+ , CD27+ , CD28+ , CD161+ ). We also identified and annotated other major cell types such as TCRγδ cells (γδ-T; CD3+ , CD4- , CD8low, TCRγδ+ ), 5 clusters of B cells (B Naive; CD19+ , CD20+ , IgD+ , CD27- ), (B memory; CD19+ , CD20+ , IgD- , CD27+ ), (B plasma; CD19+ , CD20- , CD27+ ), (B activated cells/ atypical B cells; CD19+ , CD20+ , CD11c+ ), (B activated CXCR5+ cells; CD19+ , CD20+ , CXCR5+ ) and 4 clusters of NK cells (Early-NK; CD56+CD16-), (Late-NK; CD56+, CD16+ ), (CD56+CD57+CD16- NK cells; CD45RA+ , CD38+ ), and (CD56+CD57+CD16+ NK cells; CD45RA+ , CD38+ ). Furthermore, cells of the myeloid lineage clustered into 4 subsets that were annotated as MONO classical (Classical/Intermediate Monocytes; CD11c+ , CD14+ , CD16-/lo, HLADR+), MONO non classical (non-classical monocytes; CD11c+, CD14- , CD16+ , HLADR+ ), pDCs (plasmacytoid Dendritic Cells; HLADR+ , CD123+ , CD11c- , CD38+ ) and mDCs (myeloid Dendritic Cells; HLADR+ , CD123- , CD11c+ , CD38+ ).


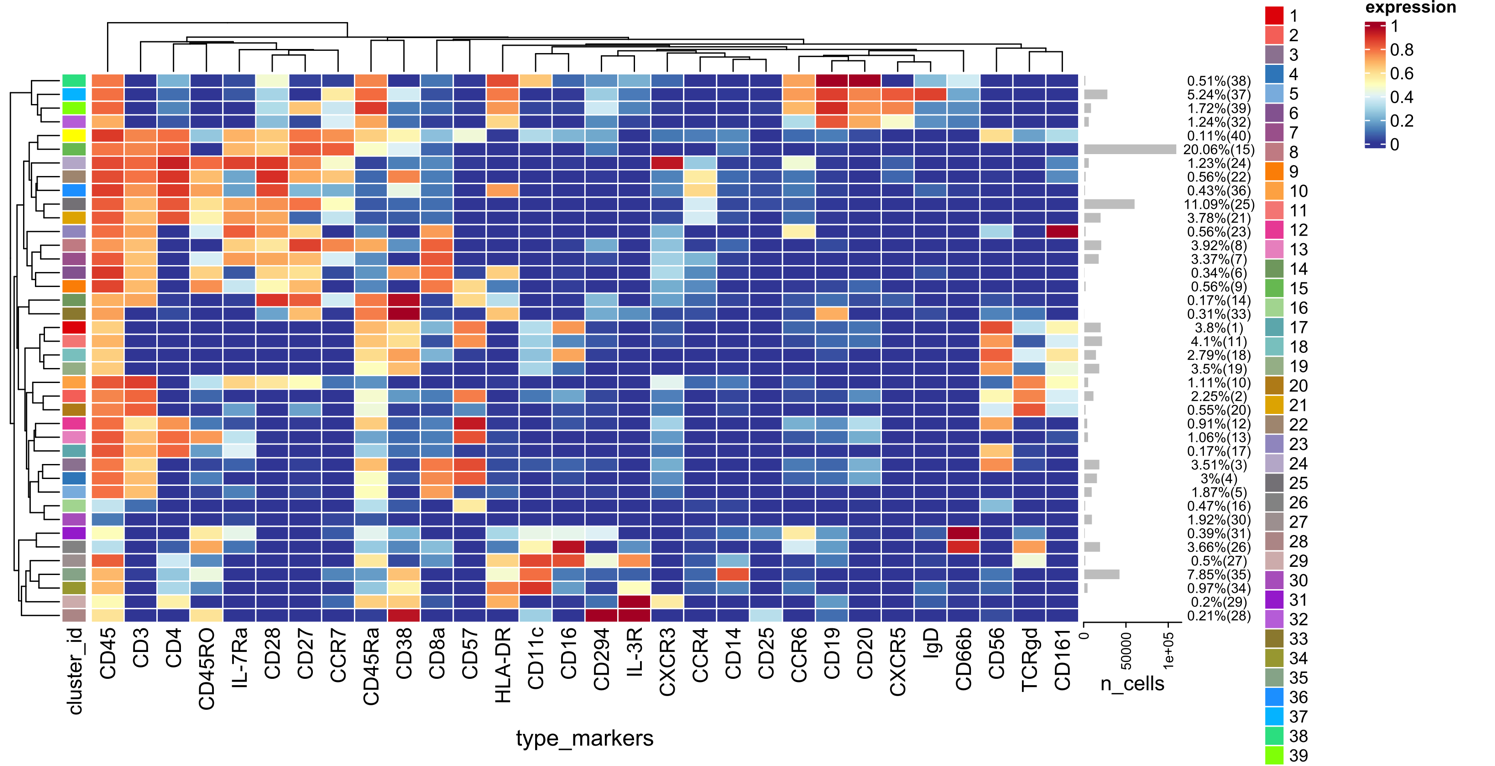


**Supplementary Figure 1.** Heatmap of the median marker intensities (arcsinh, transformed median marker expression calculated over cells from samples analysed with CyTOF, n=55) of the 30 markers across the 40 cell populations obtained with FlowSOM. Hierarchical similarity between the 40 metaclusters is noted with a dendrogram on the left (Euclidean distance; average linkage). The cluster number is shown in the parenthesis on the right.

Supplementary Table 2. List of annotated immune cell subphenotypes by mass cytometry according to the heatmap presented in Supplementary Figure 1. The number of each cluster refers to cluster number in Supplementary Figure 1.

| Subpopulation | No of Annotated cluster | Subpopulation | No of Annotated cluster |
| --- | --- | --- | --- |
| CD4 |  | NK |  |
| CD4 naïve cells | 15 | NK CD56+ CD57+ CD16+ | 1 |
| CD4 central mem | 22 | NK CD56+ CD57+ CD16- | 11 + 12 |
| CD57+ T4 cells | 13 | NK CD56+ CD57- CD16+ | 18 |
| CD56+ T4 cells | 17 | NK CD56+ | 19 |
| CD4 term eff | 21 | B cells |  |
| Th1 like | 24 | Bactiv CXCR5+ | 32 |
| CD4 eff mem | 25 | B plasmablasts | 33 |
| HLADR+ CD4 T cells | 36 | B naïve | 37 |
| TCRgd | 2 + 10 + 20 | B active | 38 |
| CD8 |  | B memory | 39 |
| CD56+ CD57+ T8 Cells | 3 | Monocytes |  |
| CD57+ T8 Cells | 4 | classical Monocytes | 35 |
| CD8 TEMRA | 5 | non-classical Monocytes | 27 |
| CD 8 HLADR+ CD38+ | 6 | DCs |  |
| CD8 effector | 7 | pDCs | 29 |
| CD8 naïve | 8 | mDCs | 34 |
| CD8 CD57+ | 9 |  |  |
| NKT cells | 23 |  |  |


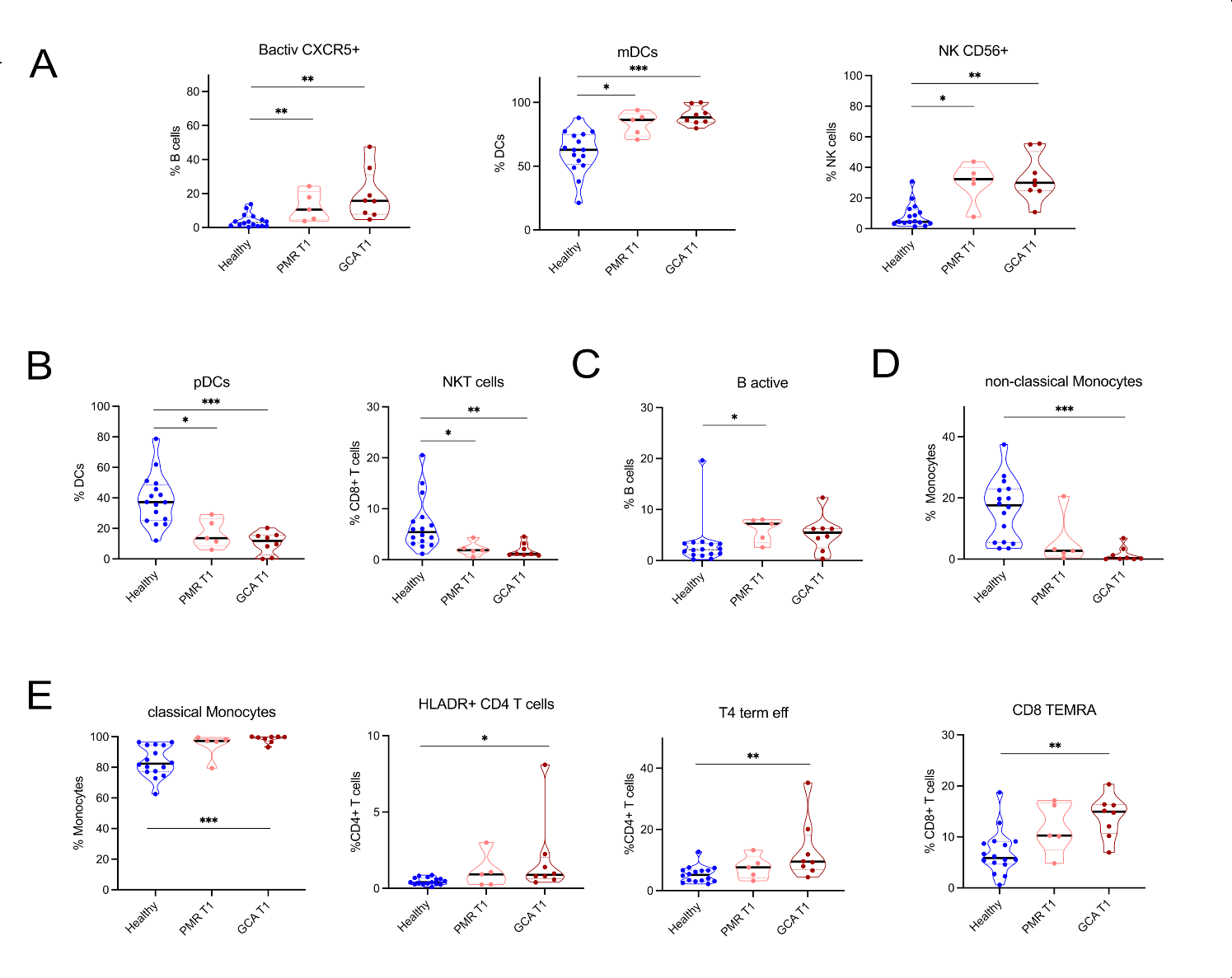


**Supplementary Figure 2. Statistically significant alterations of immune cell subsets/ subphenotypes frequencies between healthy individuals and the two disease groups at T1**. **A.** Immune cell subpopulations that exhibited increased frequencies at T1 in both PMR and GCA patients compared to healthy individuals (T1: before steroid administration). Violin plots of statistically significant alterations in CXCR5+ B active cells, mDCs, and CD56+ NK cells. **B.** Immune cell subpopulations that exhibited decreased frequencies at T1 in both PMR and GCA patients compared to healthy individuals. Violin plots of statistically significant alterations in pDCs, and NKT cells. **C.** Immune cell subpopulations that exhibited increased frequencies at T1, only in PMR patients. Violin plots of statistically significant alterations in B active cells. **D.** Immune cell subpopulations that exhibited decreased frequencies at T1, only in GCA patients, compared to healthy individuals. Violin plots of statistically significant alterations in non-classical monocytes. **E.** Immune cell subpopulations that exhibited increased frequencies at T1, only in GCA patients, compared to healthy individuals. Violin plots of statistically significant alterations in classical monocytes, HLADR+ CD4+ T cells, CD4 terminal effector cells, and CD8 TEMRA cells. Statistical comparisons of peripheral blood immune cell subset frequencies between the three groups (PMR, GCA, and Healthy controls) were performed at T1 using the Kruskal–Wallis test. When a statistically significant overall difference was observed (p < 0.05), post hoc pairwise comparisons were conducted between groups using Dunn’s test with Bonferroni correction to adjust for multiple testing. P-values reported reflect the adjusted post hoc p-values. All statistical analyses were performed independently for each of the 40 immune cell subsets. The first quartile and the third quartile are shown in the lower and upper horizontal lines, respectively. The horizontal line in the boxes represents the median value. NKT, Natural Killer T-like cells; CD, cluster of differentiation; DCs, dendritic cells; HLA-DR, Human Leukocyte Antigen – DR isotype; TEMRA, terminally differentiated effector memory cells.

Supplementary Table 3. Median frequencies of immune cell types and sub-phenotypes detected in all patients (PMR and GCA) before steroid administration (T1) (n=14), at 48 hours (T2) (n=14) and 96 hours (T3) (n=13) after steroid administration. Median frequencies are also presented for the healthy control group (n=16).

| **Subpopulation** | **T1** | **T2** | **T3** | **HC** |
| --- | --- | --- | --- | --- |
| **CD4 (% PBMCs)** | 40,19 (35,67-50,14) | 39,87 (28,23-52,56) | 38,57 (30,04-51,79) | 39,33 (33,59-46,36) |
| CD4 naïve cells (%CD4+ cells) | 45,59 (32,85-60,08) | 60,38 (43,78-69,95) | 52,47 (41,69-58,64) | 43,2 (38,4-57,58) |
| CD4 central mem (%CD4+ cells) | 1,08 (0,66-1,87) | 1,06 (0,68-1,73) | 0,99 (0,59-1,74) | 1,29 (0,97-1,93) |
| CD57+ T4 cells (% CD4+ cells) | 3,11 (1,09-4,68) | 2,8 (0,95-4,31) | 1,91 (1,21-4,15) | 0,78 (0,23-2,55) |
| CD56+ T4 cells (% CD4+ cells) | 0,36 (0,16-0,88) | 0,27 (0,13-1,21) | 0,36 (0,16-0,65) | 0,15 (0,01-0,33) |
| CD4 term eff (% CD4+ cells) | 8,93 (5,89-12,54) | 9,56 (5,02-13,14) | 14,78 (7,61-24,06) | 5,15 (3,16-6,67) |
| Th1 like (% CD4+ cells) | 0,42 (0,09-1,77) | 0,19 (0,06-0,47) | 0,14 (0,04-0,44) | 10,24 (0,27-23,98) |
| CD4 eff mem (% CD4+ cells) | 33,79 (24,77-43,69) | 22,26 (17,75-36,51) | 25,3 (16,86-35,95) | 31,19 (23,67-35,83) |
| HLADR+ CD4 T cells (% CD4+ cells) | 0,92 (0,49-1,82) | 1,5 (0,66-2,43) | 0,79 (0,39-2,603) | 0,4 (0,28-0,67) |
| **TCRgd (% PBMCs)** | 1,67 (1,16-4,53) | 1,52 (0,9-3,99) | 2,18 (0,87-4,74) | 1,78 (1,31-4,76) |
| **CD8 (% PBMCs)** | 16,16 (13,29-20,35) | 13,22 (11,5-17,16) | 14,74 (9,71-18,01) | 22,67 (19,42-28,25) |
| CD56+ CD57+ T8 Cells (% CD8+ cells) | 14,13 (6,31-18,04) | 17,71 (6,78-32,83) | 11,49 (4,34-19,99) | 9,98 (3,3-17,57) |
| CD57+ T8 Cells (% CD8+ cells) | 24,1 (9,38-40,66) | 21,12 (10,43-27,36) | 16,26 (9,19-32,74) | 7,46 (4,6-14) |
| CD8 TEMRA (% CD8+ cells) | 14,76 (10,16-16,29) | 11,47 (8,7-15,68) | 11,84 (7,13-17,54) | 5,87 (4,67-9,01) |
| CD 8 HLADR+ CD38+ (% CD8+ cells) | 2,29 (0,63-4,2) | 2,47 (1,05-4,58) | 1,58 (1,08-3,56) | 0,89 (0,68-1,42) |
| CD8 effector (% CD8+ cells) | 17,16 (12,02-27,58) | 20,89 (10,66-27,2) | 23,89 (14,85-27,51) | 20,57 (11,47-36,28) |
| CD8 naïve (% CD8+ cells) | 19,96 (6,37-27,69) | 13,42 (8,09-30,58) | 14,38 (6,36-27,88) | 28,18 (13,83-56,96) |
| CD8 CD57+ (% CD8+ cells) | 3,26 (1,54-5,49) | 2,84 (1,1-4,64) | 4,11 (1,48-5,02) | 3,17 (2,23-3,67) |
| NKT cells (% CD8+ cells) | 1,69 (0,99-2,74) | 1,5 (0,62-2,19) | 1,25 (0,81-1,74) | 5,41 (3,29-8,14) |
| **NK (% PBMCs)** | 15,01 (10,16-21,21) | 10,47 (6,75-26) | 10,37 (6,32-25,95) | 13,47 (7,85-23,66) |
| NK CD56+ CD57+ CD16+ (% NK cells) | 12,81 (5,314-22,07) | 14,46 (4,42-22,34) | 5,28 (2,87-25,71) | 48,74 (41,01-59,64) |
| NK CD56+ CD57+ CD16- (% NK cells) | 32,31 (21,59-41,44) | 36,87 (20,47-49,09) | 33,83 (23,29-47,97) | 0,13 (0,09-1,22) |
| NK CD56+ CD57- CD16+ (% NK cells) | 9,19 (4,58-17,65) | 6,14 (4,12-19,8) | 8,26 (2,34-19,06) | 35,28 (30,03-42,38) |
| NK CD56+ (% NK cells) | 31,48 (24,84-40,09) | 29,83 (20,43-45,51) | 31,63 (25,45-39,43) | 4,58 (3,56-12,32) |
| **B cells (% PBMCs)** | 7,66 (5,89-10,21) | 11,78 (8,32-13,79) | 10,06 (7,17-13,88) | 7,39 (5,71-9,91) |
| Bactiv CXCR5+ (% B cells) | 15,56 (6,39-21,66) | 11,84 (7,04-22,62) | 17,19 (7,68-26,37) | 3,04 (1,01-6,12) |
| B plasmablasts (% B cells) | 1,62 (1,02-3,91) | 2,32 (1,13-5,73) | 2,3 (1,53-8,12) | 0,85 (0,33-1,65) |
| B naïve (% B cells) | 57,47 (41,66-64,53) | 59,34 (45,63-71,92) | 55,46 (43,76-66,42) | 69,49 (54,74-73,69) |
| B active (% B cells) | 6,21 (3,48-7,54) | 6,04 (4,24-9,99) | 5,69 (3,22-6,38) | 2,13 (1,16-3,33) |
| B memory (% B cells) | 19,98 (13,28-28,02) | 12,18 (5,6-20,86) | 15,67 (5,13-23,65) | 25,08 (15,61-31,65) |
| **Monocytes (% PBMCs)** | 6,72 (3,51-10,43) | 8,91 (4,73-13,95) | 4,66 (3,32-12,95) | 7,917 (5,96-11,27) |
| classical Monocytes (% Monocytes) | 98,75 (96,69-99,85) | 99,66 (96,34-99,9) | 99,66 (98,62-99,79) | 82,4 (77,14-94,6) |
| non-classical Monocytes (% Monocytes) | 1,25 (0,15-3,31) | 0,34 (0,1-3,66) | 0,34 (0,21-1,38) | 17,6 (5,4-22,86) |
| **DCs (% PBMCs)** | 1,48 (0,37-2,54) | 0,84 (0,24-1,37) | 0,81 (0,47-1,19) | 0,84 (0,67-1,31) |
| pDCs (%DCs) | 13,6 (7,2-17,95) | 7,14 (5,13-12,88) | 4,05 (1,65-12,22) | 37,2 (25,28-48,51) |
| mDCs (%DCs) | 86,4 (82,05-92,8) | 92,86 (87,12-94,87) | 95,95 (87,78-98,35) | 62,8 (51,49-74,72) |

**Supplementary Table 4.** Median serum levels of 21 cytokines and chemokines in patients with GCA, PMR, and Healthy controls, assessed by Luminex Assay. Cytokine/chemokine levels were measured in serum samples from Healthy individuals (n = 10), GCA patients (n = 8), and PMR patients (n = 6) at baseline (T1, before glucocorticoid treatment) and at 96 hours post-treatment (T3). Median values are presented with interquartile ranges in parentheses. Statistical comparisons between groups (GCA, PMR, and Healthy) at T1(before treatment initiation) were performed using the Kruskal–Wallis test. Where significant differences were detected (p < 0.05), post hoc pairwise comparisons were conducted using Dunn’s test with Bonferroni correction for multiple testing. No statistically significant differences were observed between the GCA and PMR groups. Therefore, asterisks indicate only the comparisons between either the GCA or PMR groups and the Healthy control group (*p < 0.05, **p < 0.01, ***p < 0.001). Abbreviations: IL, Interleukin; TNF-α, Tumor Necrosis Factor alpha; IFN-γ, Interferon gamma; GM-CSF, Granulocyte-Macrophage Colony-Stimulating Factor; MIP, Macrophage Inflammatory Protein (MIP-1a, MIP-1b, MIP-3a); ITAC, Interferon-Inducible T Cell Alpha Chemoattractant.

| **Analyte** | **Healthy Individual** | **GCA T1** | **GCA T3** | **PMR T1** | **PMR T3** |
| --- | --- | --- | --- | --- | --- |
| **IFN-γ** | 12,5 (8,88-18,6) | 20,7 (17,8-31,6) | 27,8 (21-33,4) | 17,4 (5,16-37,1) | 24,9 (8,98-29,5) |
| **IL-1β** | 0,43 (0,24-0,56) | 0,54 (0,41-0,9) | 0,5 (0,21-0,8) | 0,5 (0,22-1,3) | 0,59 (0,4-1,6) |
| **IL-2** | 0,42 (0,26-0,57) | 0,67 (0,31-1,4) | 1 (0,57-1,5) | 0,9 (0,31-2) | 0,49 (0,2-1,3) |
| **IL-4** | 5,3 (2,22-12,3) | 13,1 (4,55-17,6) | 9,48 (6,07-17,2) | 15,4 (7,94-229) | 14,6 (13,5-508) |
| **IL-5** | 0,35 (0,23-1,1) | 0,85 (0,41-1,2) | 0,96 (0,49-1,4) | 0,49 (0,46-3,4) | 0,49 (0,49-5,8) |
| **IL-6** | 2,17 (1,15-3,46) | **14,19 (12,43-19,76)**** | 13,68 (11,7-17,63) | **11,28 (9,27-15,13)**** | 12,18(10,43-12,5) |
| **IL-7** | 1,4 (0,72-2,8) | **5,65 (5,18-6,87)***** | 6,63 (5,74-7,98) | **5,33 (3,68-12,8)**** | 6,01 (3,62-18,1) |
| **IL-8** | 4,21 (3,2-19,4) | **1,79 (1,18-2,6)*** | 0,855 (0,355-3,22) | 3,15 (2,28-26,9) | 4,76 (1-135) |
| **IL-10** | 2,38 (1,37-3,95) | 1,46 (1,46-5,56) | 6,39 (3,9-10,5) | 4,48 (1,43-33,4) | 5,51 (1,46-51,3) |
| **IL-12(p70)** | 0,79 (0,4-1,8) | 1,1 (0,59-2,4) | 1,6 (0,77-2,4) | 0,85 (0,54-1,7) | 0,94 (0,49-1,6) |
| **IL-13** | 0,81 (0,21-1,3) | 0,97 (0,31-2,5) | 0,73 (0,24-2,8) | 2,1 (0,24-20) | 2,5 (0,24-35) |
| **IL-17A** | 4,19 (2,12-8,9) | 5,34 (3,47-7,81) | 5,66 (4,41-7,48) | 4,15 (2,27-12,2) | 4,81 (3,5-9,73) |
| **IL-21** | 1,2 (0,7-1,6) | **0,36 (0,18-0,43)*** | 0,41 (0,26-0,61) | 0,58 (0,27-0,8) | 0,4 (0,19-0,53) |
| **IL-23** | 70,5 (40,1-134) | 31,7 (7,93-61,3) | 41,6 (9,25-58,5) | 85,3 (57,9-175) | 79,7 (25,4-197) |
| **ITAC** | 13,3 (7,01-15,1) | **34,5 (20,8-47,6)**** | 28,3 (17,1-48,7) | **50,7 (23,8-67,5)**** | 25 (16,2-50,1) |
| **GM-CSF** | 2,41 (1,11-5,21) | 4,73 (1,9-10,5) | 5,75 (2,88-7,44) | 4,82 (3,08-6,04) | 5,03 (3,09-6,94) |
| **Fractalkine** | 28,2 (24-41,1) | 22,8 (18,7-29,8) | 27,3 (21,9-30,8) | 27 (19-44,4) | 24,5 (13,9-26,6) |
| **MIP-1a** | 4,85 (1,32-8,29) | 7,98 (4,9-9,69) | 8,57 (6,05-9,7) | 10,9 (6,97-13) | 8,64 (6,34-12,3) |
| **MIP-1b** | 17,6 (14,1-21) | 10,3 (9,56-14,3) | 11,2 (8,68-16,2) | 19,3 (15,5-24,7) | 19,6 (14,5-24,3) |
| **MIP-3a** | 3,03 (1,88-3,98) | **5,6 (4,14-7,61)*** | 6,43 (4,63-7,71) | 5,43 (4,52-8) | 5,4 (4,36-6,58) |
| **TNF-α** | 2,66 (2,39-3,21) | **5 (4,58-6,26)*** | 5,06 (3,09-7,78) | **6,21 (4,58-7,05)**** | 5,53 (4,41-6,45) |


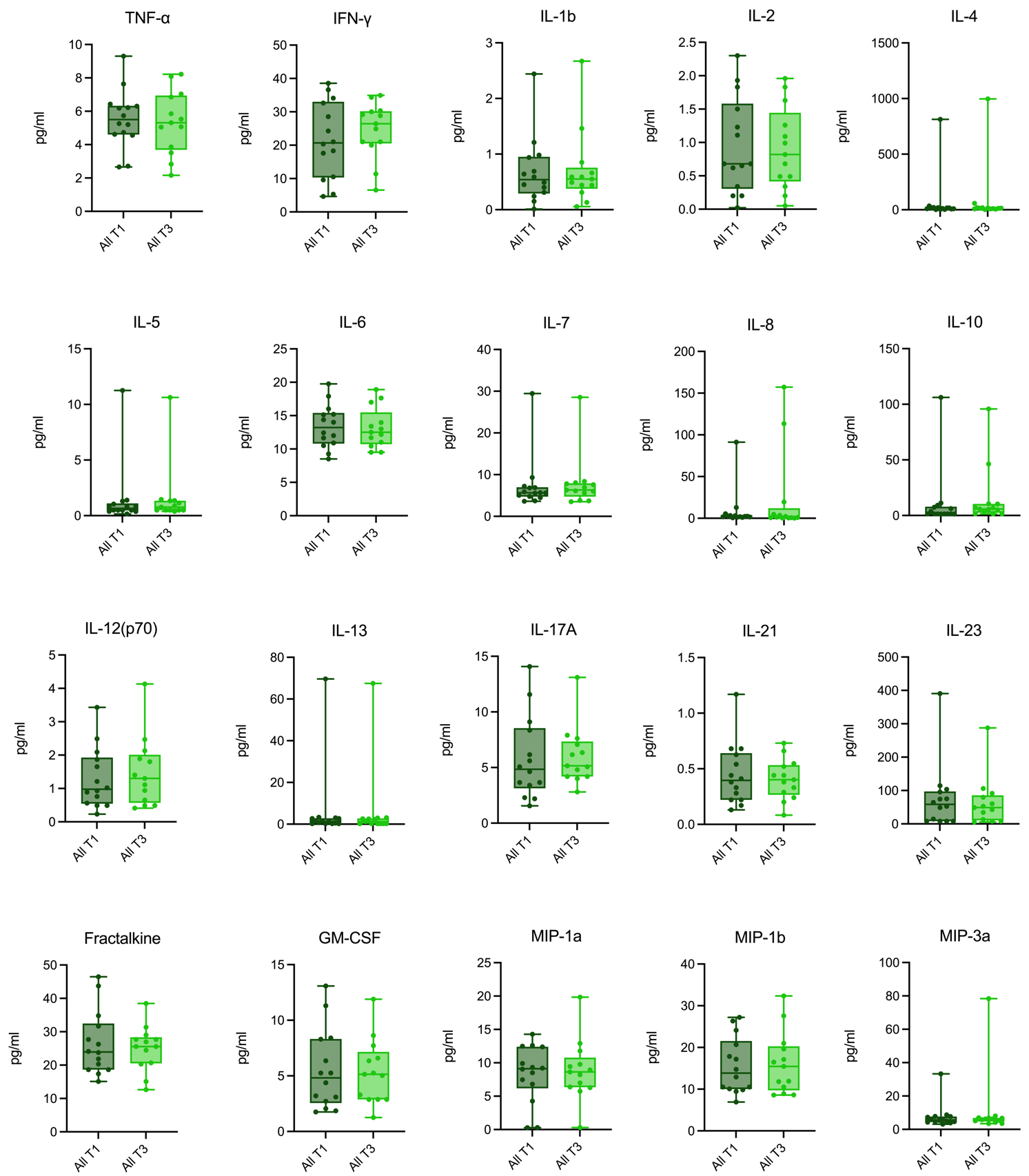


**Supplementary Figure 3.** Serum concentrations of 20 cytokines and chemokines in all patients (PMR and GCA pooled) before (T1) and 96 hours after (T3) GC treatment. Paired comparisons between time points were performed using the Wilcoxon matched-pairs signed-rank test. Only ITAC (shown separately in Figure 4C) demonstrated a statistically significant decrease following GC treatment. The graphs presented here display the cytokines and chemokines that did not show statistically significant changes between time points. Abbreviations: IL, Interleukin; TNF-α, Tumor Necrosis Factor alpha; IFN-γ, Interferon gamma; GM-CSF, Granulocyte-Macrophage Colony-Stimulating Factor; MIP, Macrophage Inflammatory Protein (MIP-1a, MIP-1b, MIP-3a); ITAC, Interferon-Inducible T Cell Alpha Chemoattractant.


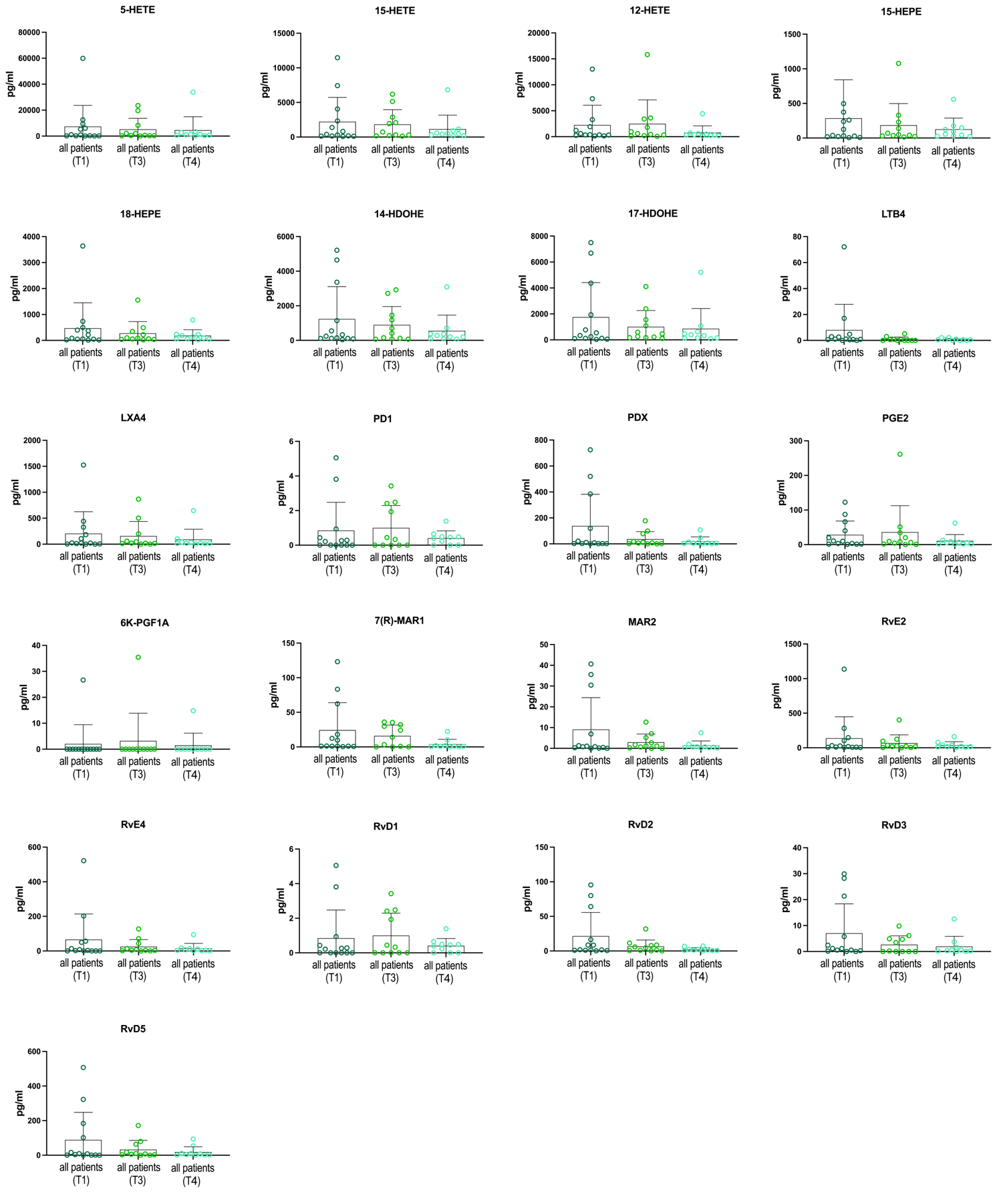
**Supplementary Figure 4.** Lipid mediators’ levels in GCA and PMR patients. Plasma levels of lipid mediators (LMs) derived from AA, EPA and DHA metabolism from PMR (n=6) and GCA (n=7) patients (total, n=13) during active phase (T1), after 48 hours (T3) and 6 months of GC treatment (T4). Data are presented as box plots with dots showing individual patient measurements. P-values were determined by Mann-Whitney t-test for non-parametric comparisons. **P*<0.05, ***P*<0.01, ****P*<0.001. AA, Arachidonic Acid; EPA, Eicosapentaenoic Acid; DHA, Docosahexaenoic acid; GC, glucocorticoids; HDoHE, Hydroxydocosahexanoic acid; HEPE, hydroxy-eicosapentaenoic acid; HETE, Hydroxy-eicosatetraenoic acid; LTB4, Leukotriene B4; LXA4, Lipoxin A4; LXB4, Lipoxin B4; 6K-PGFIA, 6-keto-prostaglandin F1 alpha; PGE2, Prostaglandin E2; Rv, Resolvin E/D series; TXB2, Thromboxane B2.
